# CHLH/GUN5 Function in Tetrapyrrole Metabolism Is Correlated with Plastid Signaling but not ABA Responses in Guard Cells

**DOI:** 10.3389/fpls.2016.01650

**Published:** 2016-11-07

**Authors:** Harue Ibata, Akira Nagatani, Nobuyoshi Mochizuki

**Affiliations:** Department of Botany, Graduate School of Science, Kyoto UniversityKyoto, Japan

**Keywords:** GUN5, CHLH, Mg-chelatase, tetrapyrrole, plastid signal, stomatal ABA response

## Abstract

Expression of *Photosynthesis-Associated Nuclear Genes* (*PhANGs*) is controlled by environmental stimuli and plastid-derived signals (“plastid signals”) transmitting the developmental and functional status of plastids to the nucleus. *Arabidopsis genomes uncoupled* (*gun*) mutants exhibit defects in plastid signaling, leading to ectopic expression of *PhANGs* in the absence of chloroplast development. *GUN5* encodes the plastid-localized Mg-chelatase enzyme subunit (CHLH), and recent studies suggest that CHLH is a multifunctional protein involved in tetrapyrrole biosynthesis, plastid signaling and ABA responses in guard cells. To understand the basis of CHLH multifunctionality, we investigated 15 *gun5* missense mutant alleles and transgenic lines expressing a series of truncated CHLH proteins in a severe *gun5* allele (*cch*) background (tCHLHs, 10 different versions). Here, we show that Mg-chelatase function and plastid signaling are generally correlated; in contrast, based on the analysis of the *gun5* missense mutant alleles, ABA-regulated stomatal control is distinct from these two other functions. We found that none of the tCHLHs could restore plastid-signaling or Mg-chelatase functions. Additionally, we found that both the C-terminal half and N-terminal half of CHLH function in ABA-induced stomatal movement.

## Introduction

CHLH (also called GENOMES UNCOUPLED 5, GUN5) is a multifunctional protein involved in chlorophyll biosynthesis, plastid-to-nucleus signaling, and ABA responses. CHLH is a plastid-localized protein that has been studied as the largest (H) subunit of Mg-chelatase, which catalyzes the conversion of Protoporphyrin IX (Proto) to Mg-Protoporphyrin IX (MgProto), a key step in the tetrapyrrole biosynthesis pathway in plants (Tanaka et al., 2011). Mg-chelatase is composed of three catalytic subunits, CHLH/GUN5, CHLD, and CHLI, and a regulatory subunit, GUN4. CHLH is the porphyrin binding subunit, and CHLD-CHLI confers Mg-ATPase activity (Gibson et al., 1995; Willows et al., 1996). CHLH protein sequences are well conserved from photosynthetic bacteria to land plants. In addition, the protein structure of CHLH has been studied using single-particle reconstruction and small-angle X-ray scattering (Qian et al., 2012), and the crystal structure of *Synechocystis* PCC6803 CHLH (SynCHLH) was recently solved at 2.5 Å resolution (Chen et al., 2015). SynCHLH is thought to consist of six domains (I–VI, Supplementary Figures 1, 14), with small amino (N)-terminal domains (domains I and II) that form a “head” and “neck” structure, followed by a cage-like assembly (domains III-VI). It was reported that CHLH predominantly exists as a monomer in solution (Qian et al., 2012), whereas a loosely bound CHLH dimer was observed in the crystal. The dimerization interface are domains I and V, which is consistent with a previous study that removal of the N-terminal 159 residues of *T. elongates* ChlH facilitates a monomeric state (Adams et al., 2014). The porphyrin-binding internal pocket is proposed to be located at the interface between domains III and V, a region with the most conserved residues. The *Arabidopsis* mutants *cch* and *gun5*−*1* have respectively a Pro to Leu substitution at residue 642 (P642L) and an Ala to Val substitution at residue 990 (A990V). These positions map to P595 and A942 in the SynCHLH protein (Supplementary Figure 1), which are located respectively in domain III and at the junction between domains III and V (Chen et al., 2015). Because the *cch* and *gun5*−*1* mutant CHLH proteins can bind Proto but are catalytically inactive, it has been proposed that these mutations may introduce spatial hindrance and interfere with chelation (Davison and Hunter, 2011).

Plastid-to-nucleus retrograde signaling (also called “plastid signaling”) controls diverse aspects of cellular activity such as plastid development, response to abiotic stress, hormone signaling, and shoot and fruit development (Chan et al., 2016). Several different signals and pathways have been identified, including tetrapyrroles, isoprenes, phosphoadenosines, carotenoid derivatives, reactive oxygen species, and proteins. The GENOMES UNCOUPLED (GUN) pathway is triggered by the arrest of plastid development under photooxidative stress induced by the carotenoid biosynthesis inhibitor norflurazon (NF) or under the inhibition of plastid translation caused by lincomycin (Susek et al., 1993; Gray et al., 1995). Under such conditions, transcription of photosynthesis-associated nuclear genes (PhANGs) such as *light-harvesting chlorophyll a/b binding protein1* (*Lhcb1*) is severely repressed. Genetic studies using *Arabidopsis* have led to the discovery of *gun* mutants that exhibit derepression of Lhcb1^*^2 under these conditions (hereafter called the “*gun* phenotype”) (Susek et al., 1993; Mochizuki et al., 2001). Of six *GUN* genes, *GUN2*–*GUN6* are involved in tetrapyrrole metabolism, suggesting that tetrapyrrole is key for such signaling. *GUN2* (*HY1*), *GUN3* (*HY2*), and *GUN6* (*FC1*) encode a heme oxygenase, phytochromobilin synthase, and Fe-chelatase, respectively (Mochizuki et al., 2001; Woodson et al., 2011). As mentioned above, *GUN4* and *GUN5* encode the regulatory and porphyrin-binding subunits of Mg-chelatase, respectively (Mochizuki et al., 2001; Larkin et al., 2003). GUN1 is a plastid-localized pentatricopeptide repeat-small MutS-related protein (PPR-SMR) that is proposed to serve as a central hub of GUN plastid signaling (Koussevitzky et al., 2007). A recent report suggests that the GUN1 protein interacts with plastid ribosomal proteins and early tetrapyrrole biosynthesis enzymes (Tadini et al., 2016). Although several lines of evidence suggest that Mg-protoporphyrin IX and heme status are key in plastid signaling, the exact signaling mechanism remains elusive (Mochizuki et al., 2001, 2008; Strand et al., 2003; Moulin et al., 2008).

CHLH is also reported to be involved in responses to abscisic acid (ABA) in *Arabidopsis*. Recent studies reveal that the family containing pyrabactin resistance (PYR), pyrabactin resistance 1-like (PYL), and regulatory component of ABA receptor (RCAR) proteins are potent candidates for ABA receptors that regulate SnRK2 family protein kinases through type 2C protein phosphatases (PP2Cs) (Cutler et al., 2010). In a different experimental approach, CHLH was identified as an ABA-binding protein, and subsequent biochemical and genetic analyses suggested that CHLH binds to ABA and mediates the ABA signal in the regulation of stomatal aperture, seed germination, root elongation, and induction of ABA-responsive genes (Zhang et al., 2002; Shen et al., 2006). However, whether CHLH binds to ABA and has functions other than stomatal ABA responsiveness remain unclear (Shen et al., 2006; Muller and Hansson, 2009; Wu et al., 2009; Tsuzuki et al., 2011). In a subsequent study, CHLI1, but not CHLD and GUN4, was shown to be involved in ABA signaling resulting in stomatal movement in *Arabidopsis* and tobacco (Tsuzuki et al., 2011; Du et al., 2012). Moreover, it has been reported that knockdown of *CHLM* (encoding the enzyme responsible for the step after Mg-chelatase) also leads to ABA insensitivity in stomatal movement (Tomiyama et al., 2014). Furthermore, as a reduction in Mg-chelatase activity was observed in *CHLM* antisense RNA-overexpressing tobacco (Alawady and Grimm, 2005), defects in the CHLM protein may affect the function of the Mg-chelatase complex. These findings suggest that Mg-chelatase and CHLH are involved in the stomatal response to ABA.

Structural and functional studies have been carried out in *Arabidopsis* to elucidate the molecular nature of CHLH in ABA signaling (Wu et al., 2009; Shang et al., 2010). It has been proposed that the CHLH protein spans the outer and inner envelopes, with the N- and C-termini exposed to the cytoplasm (Wu et al., 2009). A group of WRKY transcription repressors are suggested to interact with the cytoplasmic C-terminal region of CHLH (Shang et al., 2010). When CHLH binds to ABA, WRKY proteins are recruited to CHLH and ABA-responsive genes are induced in the nucleus. For these experiments, transgenic *Arabidopsis* lines expressing truncated CHLH in wild type and the *cch* mutant background were produced and tested for sensitivity to ABA. With regard to ABA sensitivity in stomatal movement, both CHLH fragments with an internal deletion (line C370, a.a. 1–120 + 631–999; full-length CHLH is 1381 a.a.) and C- and N-terminally truncated CHLH (a.a. 1–772 and 631–1381, respectively) conferred ABA responsiveness. The authors concluded that the C-terminal half of CHLH plays a central role in ABA signaling and that the N-terminal region is required for proper regulation of the C-terminal half. It should be noted that line C370 complemented tetrapyrrole and chlorophyll synthesis deficiencies in the *cch* mutant.

New candidates for the signaling components downstream of CHLH-mediated ABA signaling have been suggested in recent reports. By yeast two-hybrid system using a.a. 348–1038 or 692–1381 of CHLH protein as a bait, CPN20 (plastid co-chaperonin) and SnRK2.6/OST1 (sucrose non-fermenting 1 (SNF1)-related protein kinase 2 family/open stomata 1) have been found as interaction partners, respectively (Zhang et al., 2013; Liang et al., 2015). SOAR1, which encodes a pentatricopeptide repeat (PPR) protein localizing in both the cytosol and nucleus, has been identified by a suppressor screening of ABA-hypersensitive phenotype in overexpressor of the truncated (a.a. 631–999) CHLH (Mei et al., 2014). Although it has been suggested that these genes are involved in ABA signaling including the regulation of stomatal aperture, seed germination, and seedling growth, the functions in Mg-chelatase and plastid signaling have not been examined.

Despite knowledge of the detailed structure as well as functional analyses, the relationship between the function of the CHLH protein as a Mg-chelatase enzyme and component of plastid and ABA signaling is unclear to date. Because one of the difficulties in studying CHLH is that the null mutation results in an albino and seedling-lethal phenotype, we performed a new screen for *gun* mutants, isolated a number of missense mutant alleles of *gun5* and performed a detailed phenotypic analysis. To compare functionality in different physiological responses, we also produced *Arabidopsis* transgenic lines expressing a series of truncated CHLH/GUN5s based on previous works (Wu et al., 2009).

## Materials and methods

### Plant material and growth conditions

The *Arabidopsis thaliana* strains used were all derived from the *Col-0* ecotype. *gun5* mutant alleles were isolated from the transgenic line CL3–5, as described below. *gun5*−*1* and *cch* (*conditional chlorina*, Mochizuki et al., 2001) were backcrossed to CL3–5. In all the experiments, CL3–5 was used as wild type. *rtl1* (*rapid transpiration in detached leaves 1*, Tsuzuki et al., 2011) was provided by Dr. T. Kinoshita (Nagoya Univ.), and the R1290C line (in the *gl1* background) was provided by Dr. A. Takemiya (Yamaguchi Univ.) Plants were grown in soil with 1000-fold-diluted Hyponex or on Murashige and Skoog Plant Salt Mixture (Wako) agar medium containing 2% (w/v) sucrose at 23°C. For photobleaching, seedlings were grown for 5 days on MS agar medium supplemented with 2% sucrose and 2.5 μM Norflurazon (NF, Sandoz Pharmaceutical) under continuous white light (100 μmol/m^2^/s). For measurement of stomatal aperture, plants were grown on vermiculite under long-day condition [16 h fluorescent light (50 μmol/m^2^/s)/8 h dark cycle] at 23°C and approximately 85% RH. For testing inhibitory effect of ABA on seed germination and seedling growth, seeds were sown on MS agar medium containing 2% (w/v) sucrose in the absence or presence of 0.5 μM ABA, and the plates were incubated as described above after stratification.

### Production of the Lhcb1^*^2 promoter-luciferase line CL3–5

The 5′ upstream region (1.1 kb) of the Lhcb1^*^2 gene was amplified from Co-0 genomic DNA by PCR with CAB3pro-Fw0 and CAB3pro-Rv0' primers. The fragment was digested with *HindIII/NcoI* and ligated into pGL3basic (Promega), resulting in the pGL3basic/Lhcb1^*^2pro:LUC plasmid. The *HindIII-EcoRI* (blunted) region of the pBI-Hyg/35S-NosT vector (Yamaguchi et al., 1999) was inserted into the *HindIII-KpnI* (blunted) site in pCGN1547 (McBride and Summerfelt, 1990), which resulted in pCGN1547/35S-nosT. The CMV *35S* promoter was removed from pGCN1547/35S-nosT by *HindIII-XbaI* digestion and substituted with the *HindIII-XbaI* fragment of Lhcb1^*^2pro-LUC from pGL3basic/Lhcb1^*^2pro-LUC to generate pGCN/CAB3-LUC. The resulting plasmid was used for the plant transformation as described below. The primer sequences are listed in Supplementary Table 1.

### EMS treatment and mutant screening

CL3–5 seeds were treated with 0.2% (v/v) EMS at 25°C for 16 h. To obtain M2 seeds, the seeds were washed thoroughly, sown on vermiculite, and grown under continuous fluorescent light at 23°C. Approximately 66,000 M2 seeds from 20,000 M1 plants were subjected to the following screen. Individual NF-treated seedlings were submerged in 0.1 mM Luciferin (Calbiochem) and 0.01% Tween-80 in 96-well opaque titer plates. Bioluminescence was measured using a Microbeta scintillation counter (Perkin Elmer). Seedlings showing greater than 3-fold increase in luminescence compared to the average counts from the entire population was selected. By measuring luciferase luminescence in individual seedlings, we could detect mutants with a weaker phenotype than the previously reported *gun* mutants (Susek et al., 1993). The selected seedlings were transferred to MS plates to regenerate and set seeds. DNA was prepared from tissue from a portion of the M2 plants, and the genomic *GUN5* region was amplified by PCR and sequenced using the primers listed in Supplementary Table 1.

### Production of tCHLH constructs

Truncated CHLH (tCHLH) lines were produced as follows. The 5′-upstream region (1 kb) of *CHLH* was PCR amplified from Co-0 genomic DNA using primers CHLHpro-SL-1 and CHLHp3R. pGWB511 (Nakagawa et al., 2007) was amplified using primers pGWB514-SL-11 and GW500spacerF. These fragments were connected at the overlapping sequences by a SLiCE reaction (Zhang et al., 2012), resulting in pGWB511-CHLHpro. pGWB511-CHLHpro was digested with *KpnI* and used for a SLiCE reaction with the tCHLH fragments prepared as described below. The tCHLH clones listed in **Figure 5** were constructed as follows. Fragments A, B, C, D, H, I, J, and full-length were PCR amplified from the *CHLH* cDNA clone using the primers listed in Supplementary Table 1. Fragment E was produced by overlap extension PCR (Horton et al., 1989). We first amplified the 5′ fragment using CHLH-GW-Met and CHLH-120_631-Rv and the 3′ fragment by separately using CHLH-120_631-Fw and CHLH-1381-Rv. These two PCR products were then mixed and PCR amplified for 5 cycles. After the addition of primers CHLH-GW-Met and CHLH-1381-Rv, PCR was further performed for 15 cycles to obtain fragment E. Fragment E was cloned into pENTR/D-TOPO (Invitrogen), resulting in pENTR-E. Fragments E, F, and G were amplified using pENTR-E as a template. All fragments were ligated to *KpnI*-digested pGWB511-CHLHpro by a SLiCE reaction, which resulting in p511-full and p511-A through p511-J. The plasmid was used for the plant transformation as described below. The primer sequences are listed in Supplementary Table 1.

### Transformation of agrobacterium and plants

The plasmid was introduced into *Agrobacterium tumefaciens* (GV3101::pMP90) by the freeze-thaw method (Chen et al., 1994), which was subsequently transferred to plants using floral dip method (Clough and Bent, 1998). Transgenic plants were selected on MS agar medium containing the relevant antibiotics. T3 or T4 homozygous plants were used in all experiments.

### RNA extraction and RT-qPCR

Total RNA was extracted from whole seedlings using the Sepazol RNA I super kit (Nacalai Tesque) following the manufacturer's instructions. cDNA was synthesized with oligo(dT)_12–18_ using Transcriptor first-strand synthesis kit (Roche) according to the manufacturer's instructions. Real-time PCR was performed using LightCycler 480 SYBR Green I Master (Roche) and a LightCycler 96 (Roche). The following standard thermal profile was used for all reactions: 95°C for 5 min, followed by 40 cycles of 95°C for 5 s, 55°C for 10 s, and 72°C for 20 s. The primer sequences are listed in Supplementary Table 1.

### Quantification of protoporphyrin IX, mg-protoporphyrin IX, and mg-protoporphyrin IX monomethylester

Porphyrins were extracted according to Mochizuki et al. (2008), and Mg-Protoporphyrin IX (MgProto) and Mg-Protoporphyrin IX monomethylester (MgProtoMe) were detected at an excitation wavelength of 417 nm and emission at 595, nm with a 15-nm band path. After the MgProtoMe peak was eluted, the detector was adjusted for the detection of Protoporphyrin IX (Proto, excitation, 402 nm; emission 633 nm). Standard curves were constructed with authentic standards purchased from Frontier Science.

### Quantification of chlorophyll levels

Chlorophyll was extracted for 16 h in complete darkness at 4°C from 10 5-day-old seedlings using N, N-dimetylformamide. The extract was subjected to spectrophotometric measurements at 664, 647, and 750 nm. The specific chlorophyll content was calculated using the equations of Porra et al. (1989) and normalized to the total fresh weight for each sample.

### Anti-full-length CHLH antiserum production

A 6xHis-full-length CHLH protein was produced exactly as previously described (Wu et al., 2009). Briefly, cDNA encoding full-length *CHLH* was amplified by PCR using primers CHLH-Z1-F and CHLH-Z1-R. The PCR product was digested with *EcoRI* and *SalI* and then cloned into pET48b(+) at the *EcoRI* and *SalI* sites. The recombinant CHLH protein was expressed in *E. coli* Rosetta gami2 (DE3) (Novagen) and purified on a Ni^2+^-NTA column, as described in the manual provided by the manufacturer (Qiagen). Antiserum against 6xHis-full-length CHLH was raised by immunizing rabbits according to a standard protocol (Kiwa Laboratory Animals Co., Ltd). The primer sequences are listed in the Supplementary Table 1.

### Immunoblot analysis

Protein extraction from *Arabidopsis* seedlings was performed as described (Shen et al., 2006). A 10–20-μg sample of protein was separated by 7.5% SDS-PAGE and blotted onto a nitrocellulose membrane. The rabbit anti-CHLH polyclonal antibody (see above) and a mouse anti-FLAG monoclonal antibody (Wako) were used as primary antibodies at a dilution of 1:5000. An anti-rabbit IgG antibody or anti-mouse IgG antibody conjugated with horseradish peroxidase (Promega) was used as a secondary antibody at a dilution of 1:5000. Quantitation of band strength was performed using Multi Gauge Ver3.0 (FUJIFILM, Japan).

### Preparation of epidermal peels for measurement of stomatal aperture

Intact epidermal peels were prepared according to the Perforated-tape Epidermal Detachment method (the PED method, Ibata et al., 2013). For experiments, fully expanded rosette leaves were harvested from 4- to 6-week-old *Arabidopsis* plants. For PED, Time Tape (Hirasawa) was used to hold the adaxial surface of the leaf, and Scotch Magic Tape (3M) perforated with a hole was used to detach the lower abaxial epidermis from the *Arabidopsis* leaves. The epidermal peel affixed to the tape was immediately submerged in an appropriate buffer or solution for subsequent experiments.

### Measurement of stomatal aperture

Stomatal aperture was measured according to Inoue et al. (2008), with some modifications. Leaves were soaked in 3 ml 1/2 MS medium and incubated in the dark for 16 h at 23°C to cause stomatal closure. The epidermal layers were prepared by the PED method, and the layers were maintained in 3 ml of basal reaction buffer [5 mM MES-BTP (MES, 2-(N-morpholino) ethanesulfonic acid and BTP, 1,3- bis (tris (hydroxymethyl) methylamino) propane], pH 6.5, 50 μM KCl, and 0.1 mM CaCl_2_). The samples were irradiated for 2.5 h at 23°C with red light at 50 μmol/m^2^/s (Red) or blue light at 10 μmol/m^2^/s supplemented with 50 μmol/m^2^/s red light (Blue) in the absence or presence of 20 μM ABA. For ABA-induced stomatal closure, pre-illuminated epidermal tissues were incubated under blue light supplemented with red light, as described above, for 2.5 h with or without 20 μM ABA. Images of the epidermis were captured using a microscope (BX51, Olympus) equipped with a digital camera (C11440, HAMAMATSU). The images were analyzed with ImageJ (NIH) software to obtain stomatal aperture data. Only visibly intact guard cells were subjected to stomatal aperture measurement. For each strain, the size of the stomatal aperture is expressed as the mean of 25 stomata with the standard deviation (SD).

## Results

### Screening for new *gun5* missense mutant alleles

**Figure 1:**
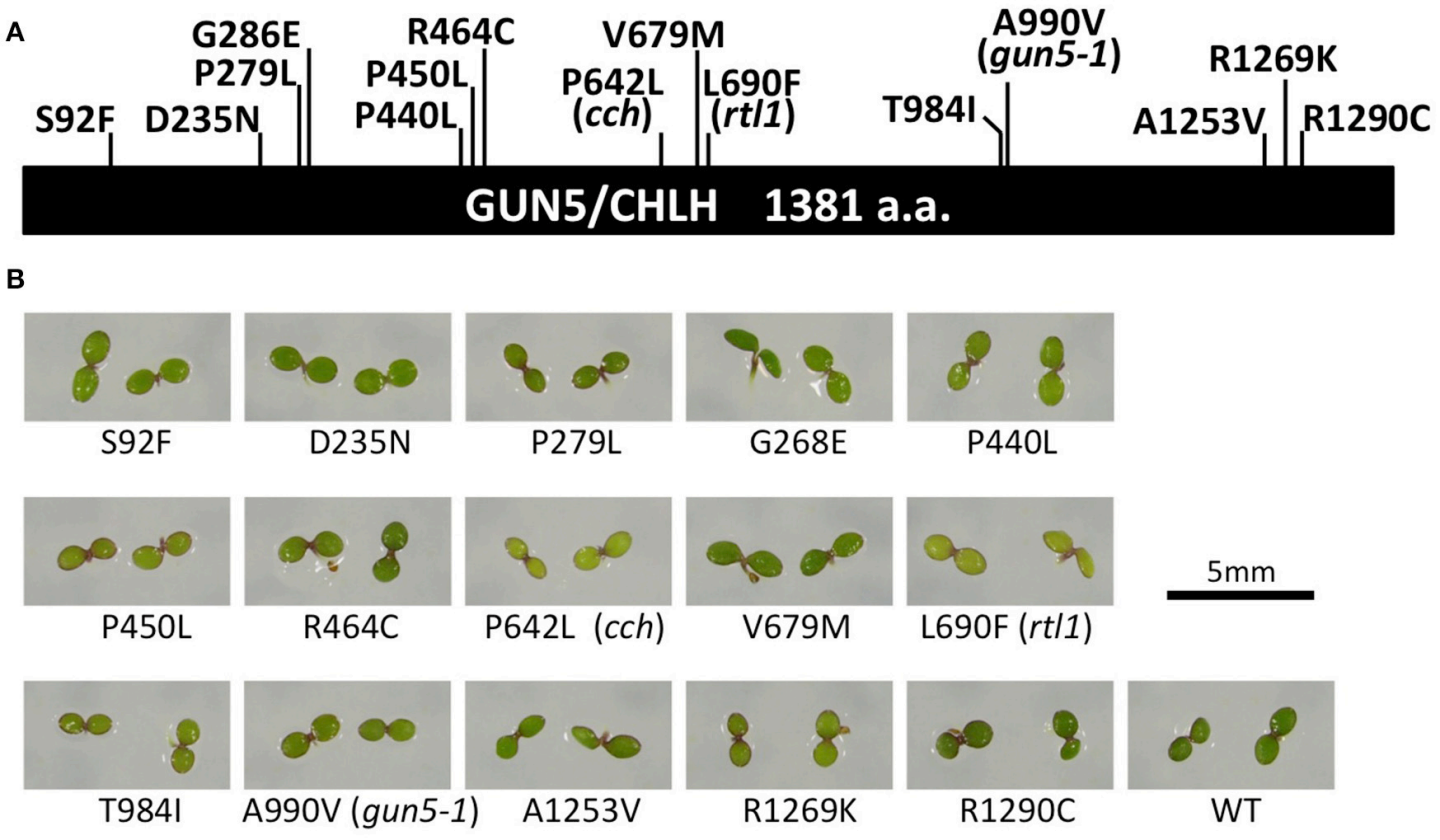
*****gun5*** mutant alleles used in this study. (A)** Schematic diagram of the GUN5/CHLH protein and the positions of amino acid substitutions of the mutant alleles used in this study. Wild type amino acids and positions and mutant amino acids are indicated. **(B)** *gun5* mutant seedlings were grown on MS medium supplemented with 2% sucrose under 100 μmol/m^2^/s white light for 5 days. The scale bar is 5 mm.

We screened for new *gun5* mutant alleles using a transgenic line harboring Lhcb1^*^2pro:*LUC* (CL3–5). We isolated 120 candidate lines showing elevated expression of the reporter in the presence of NF. Among these candidates, we identified 11 *gun5* mutant alleles by determining the genomic sequence of *GUN5/CHLH* regions (Figures 1A,B). These *gun5* alleles were backcrossed to the parental CL3–5 line at least three times and used for further analysis. A mutant (R1290C) plant that exhibits abnormal leaf surface temperature due to the opened stomatal phenotype, kindly provided by Dr. Takemiya from a mutant collection (Takemiya et al., 2013), was also examined. Three previously reported *gun5* mutant alleles (*cch, rtl1*, and *gun5*−*1*) were also included in our analysis.

In the following sections, we evaluate the mutants for three different phenotypes: (1) *gun* phenotype—Lhcb1^*^2 expression; (2) Mg-chelatase phenotype—tetrapyrrole intermediate and chlorophyll levels in 5-day-old seedlings; and (3) ABA phenotype - light-dependent change in stomatal aperture in leaves of adult plants treated with ABA.

### Analysis of plastid signaling and tetrapyrrole intermediates and chlorophyll levels in new *gun5* mutants

**Figure 2:**
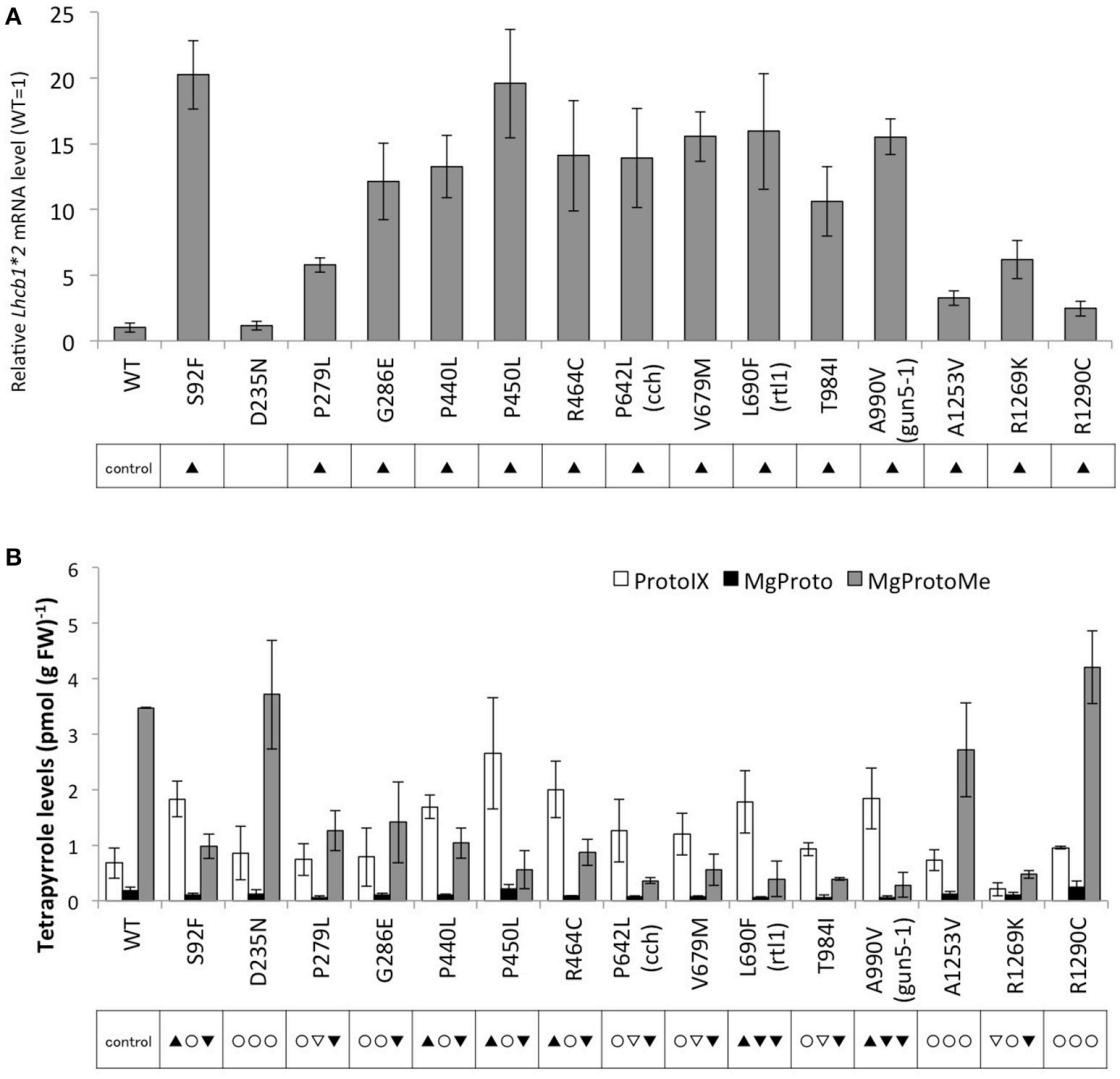
**Comparison of ***gun*** and tetrapyrrole phenotypes in wild type and ***gun5*** mutant alleles in the presence of NF**. Plants were grown on MS medium supplemented with 2% sucrose and 2.5 μM NF for 5 days under continuous white light (100 μmol/m^2^/s). **(A)** *Lhcb1* mRNA levels in wild type and *gun5* mutants grown in the presence of NF. *Lhcb1* mRNA levels were quantified and normalized to *TUB2* mRNA levels by RT-qPCR, as described in Materials and Methods. Data shown are the mean ± SD (*n* = 3), and the WT level is presented as 1.0. **(B)** Proto, MgProto, and MgProtoMe levels in wild type and *gun5* mutants in the presence of NF. Tetrapyrrole was extracted and quantified by HPLC and normalized to fresh weight, as described in Materials and Methods. Proto (white bar), MgProto (black), and MgProtoMe (gray) levels are presented. Data shown are the mean ± SD (*n* ≧ 3). In **(A)** and **(B)**, statistical significance was determined by Student's *t*-test. The black triangle and the inverted black triangle indicate significant differences (*p* < 0.05) of the average value with the higher value or lower value compared to that of the wild type control, respectively. For **(B)**, significance in Proto, MgProto, MgProtoMe are indicated from left to right in each box, respectively. The symbols are the same as **(A)**, except that the white triangle and inverted white triangle indicate weaker significant differences (*p* < 0.057) of the average value with the higher value or lower value compared to that of the wild type control, respectively, and the white circle indicates no significant difference.

For evaluation of the *gun* phenotype, endogenous Lhcb1^*^2 mRNA levels were measured by RT-qPCR. Most of the mutant seedlings exhibited elevated Lhcb1^*^2 expression in the presence of NF, though a mutation near the N-terminal region (D235N) did not result in significantly elevated Lhcb1^*^2 expression compared to wild type (Figure 2A). However, the periphery of the N-terminus of CHLH appears to be crucial for CHLH function because S92F showed a strong *gun* phenotype. We then tested tetrapyrrole intermediate levels under the same condition (+NF) and found significant correlation between Lhcb1^*^2 expression and tetrapyrrole levels (Figure 2, Supplementary Figures 3D–F). While the levels of Proto and MgProtoMe are positively and negatively correlated (*p* < 0.001) to the level of Lhcb1^*^2 expression, respectively, we found a less significant correlation between the MgProto levels and the *gun* phenotype in the presence of NF, despite MgProto levels tending to be lower in the mutants compared to the wild type (Figure 2B, Supplementary Figure 3E), and *gun* phenotype (+NF) showed significant negative correlation (*p* < 0.002) to the MgProto levels in the absence of NF (Figure 2A, Supplementary Figures 2B, 3B). Elevation of Proto and decrease of MgProto & MgProtoMe in these mutants are the results of a defect in Mg-chelatase activity, which appears to be correlated to the *gun* phenotype as proposed previously (Strand et al., 2003). However, it is evident that mutations at the C-terminus (A1253V and R1290C) resulted in a normal level of tetrapyrrole intermediate with a weak but distinct *gun* phenotype. Chl *a* levels (−NF) were tied in to the Lhcb1^*^2 mRNAs in the absence of NF (*p* < 0.05) (Supplementary Figures 2A,C, 3G), although no significant link between Chl levels and *gun* phenotypes (+NF) in the mutants were found (Supplementary Figure 3H).

### Stomatal ABA response in *gun5* mutants

**Figure 3:**
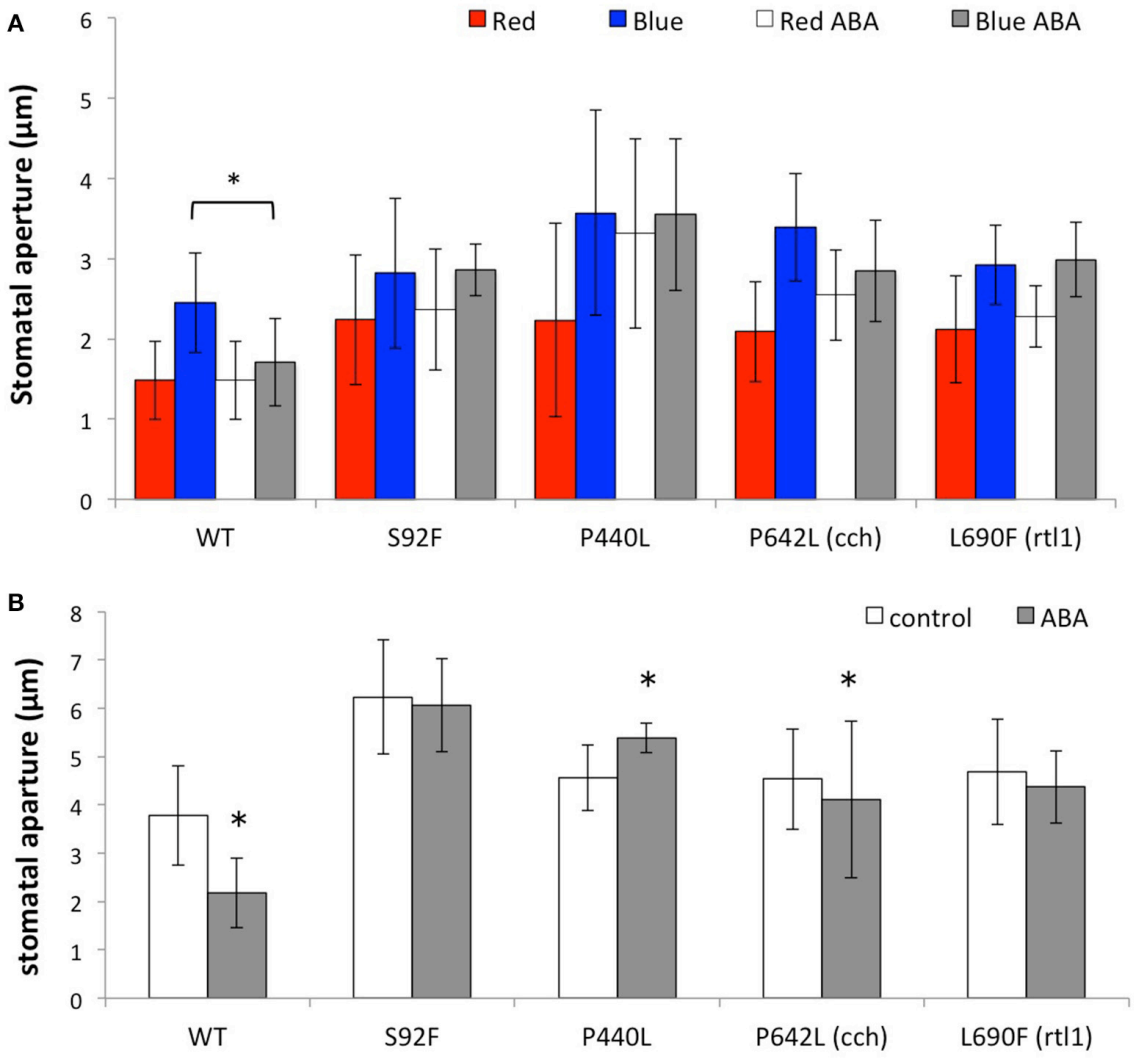
**Characterization of ABA-insensitive ***gun5*** mutants. (A)** Effect of ABA on light-induced stomatal opening in WT, S92F, P440L, P642L (*cch*), and L690F (*rtl1*) mutant plants. Epidermal tissues from dark-adapted plants were incubated under the following conditions for 2.5 h: red light at 50 μmol/m^2^/s in the absence (red bar) or presence (white bar) of 20 μM ABA (+ABA); blue light at 10 μmol/m^2^/s with background red light at 50 μmol/m^2^/s in the absence (blue) or presence (gray) of 20 μM ABA. **(B)** ABA-induced stomatal closure in wild type and mutants. Pre-illuminated epidermal tissues were incubated under blue light supplemented with red light for 2.5 h with (gray bar) without (white) 20 μM ABA. Data are presented as the mean ± SD (*n* = 25). Statistical significance was determined by Student's *t*-test. ^*^p < 0.05.

**Figure 4:**
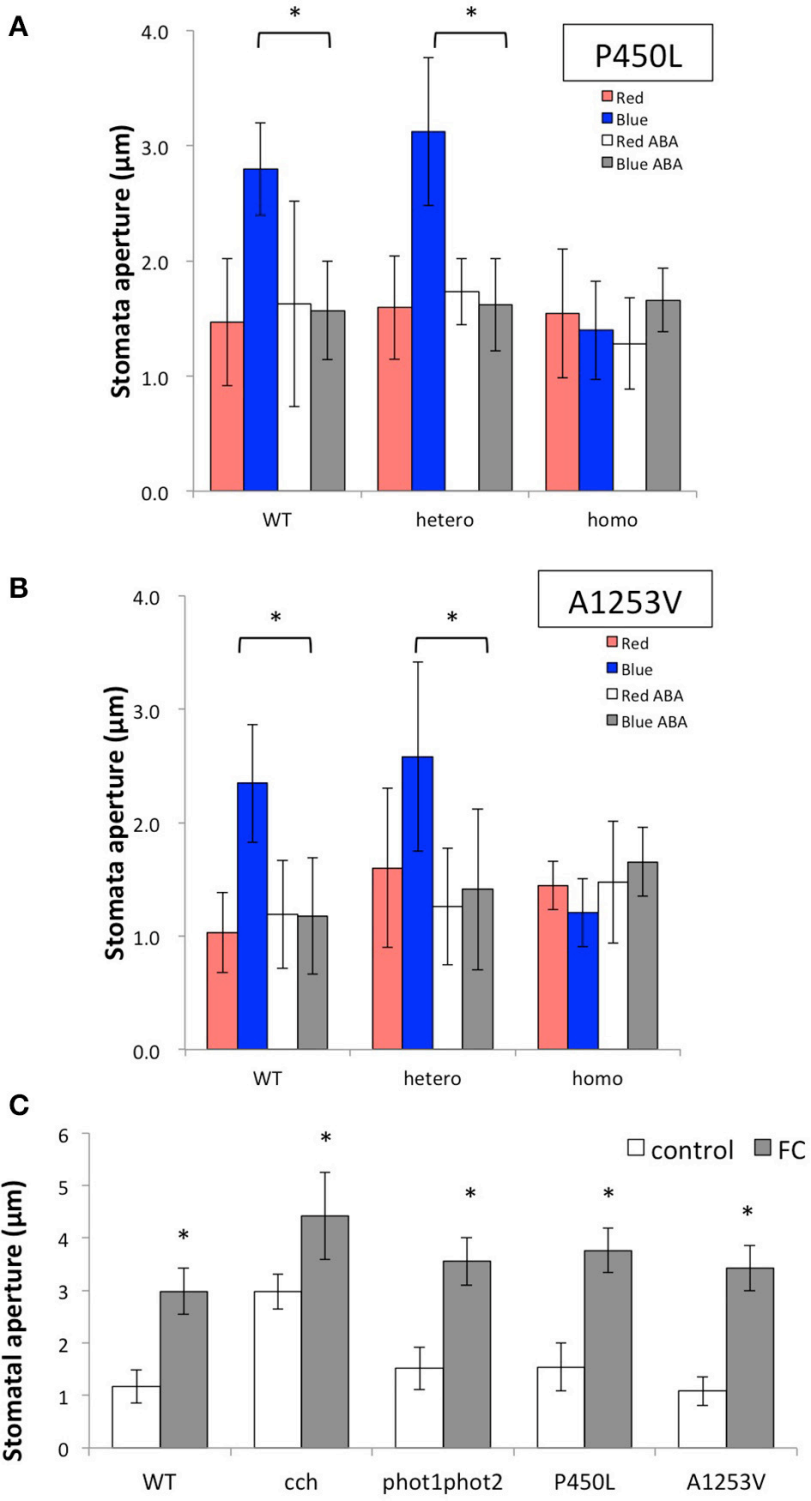
**Characterization of stomatal phenotype in P450L and A1253V mutants**. Effect of ABA on light-induced stomatal opening in backcrossed F2 progeny of **(A)** P450L and **(B)** A1253V mutants. Wild type (WT), heterozygous (hetero), and homozygous (homo) segregants were subjected to the analysis described in Figure 3A. Epidermal tissues from dark-adapted plants were incubated under the following conditions for 2.5 h: red light at 50 μmol/m^2^/s in the absence (red bar) or presence (white bar) of 20 μM ABA (+ABA); blue light at 10 μmol/m^2^/s with background red light at 50 μmol/m^2^/s in the absence (blue) or presence (gray) of 20 μM ABA. Data are presented as the mean ± SD (*n* = 25). **(C)** Effect of fusicoccin on light-induced stomatal opening in P450L and A1253V mutants. Epidermal tissues from dark-adapted plants were incubated under the following conditions for 2.5 h: blue light at 10 μmol/m^2^/s with background red light at 50 μmol/m^2^/s in the absence (white) or presence (gray) of 10 μM fusicoccin. Data are presented as the mean ± SD (*n* = 25). Statistical significance was determined by Student's *t*-test. ^*^p < 0.05.

Light-induced stomatal opening and the inhibitory effect of externally applied ABA were tested in the mutants. Plants for only four mutant alleles, including the previously reported *cch* and *rtl1*, were insensitive to ABA in close-to-open and open-to-close assays (Figures 3A,B). Most other lines (9 alleles) responded normally to ABA with regard to light-induced stomatal opening (Supplementary Figure 4). In addition to ABA-insensitive alleles, we found two mutations (P450L and A1253V) displaying a constitutively closed stomatal phenotype (Figures 4A,B, indicated as “homo”). Overexpression of CHLH in guard cells driven by an epidermis-specific promoter (*CER6*pro) is reported to exhibit a similar phenotype (Tsuzuki et al., 2013). Unlike the *CER6*pro−CHLH-GFP overexpressor, P450L and A1253V did not show a hypersensitive phenotype in response to 20 μM ABA (Figures 4A,B), and only a trace level of CHLH protein was found for the P450L mutant (Supplementary Figure 5A). Analysis of stomatal phenotype in the backcrossed F1 plants indicates that the P450L and A1253V mutations are recessive (Figures 4A,B, indicated as “hetero”). As the constitutively closed stomatal phenotype may be caused by a lack of plasma membrane-localized H^+^-ATPase activation, we treated these mutants with fusicoccin, an activator of H^+^-ATPase that induces stomatal opening without light stimulation. The stomatal apertures of the P450L and A1253V mutants were restored to wild type when treated with fusicoccin (Figure 4C), suggesting these mutants have defects in the upstream regulatory pathway leading to the activation of the stomatal plasma membrane localized-H^+^-ATPase. In view of the disparities in previous reports on other ABA-related phenotypes of *gun5* mutants (ABA resistance on seed germination and growth) (Shen et al., 2006; Muller and Hansson, 2009; Wu et al., 2009; Tsuzuki et al., 2011), we tested the *cch* and *rtl1* mutants for the seed germination and seedling growth in the presence of 0.5 μM ABA. We found equal sensitivity to ABA between the mutants and the wild type (Supplementary Figure 6). This data suggests that CHLH function in ABA-related function is limited to the stomatal guard cells.

### CHLH protein level in *gun5* mutants

Differences in phenotype among plants with the mutant alleles might be due to variation in CHLH protein accumulation and protein function. Hence, we examined CHLH protein levels in the mutants by Western blotting using an anti-CHLH antibody. CHLH is greatly reduced in wild type in the presence of NF (Supplementary Figures 5B,C) In contrast, the majority of the mutants had equivalent or even higher levels of CHLH protein than wild type in the absence or presence of NF. These results suggest that a complete absence of the CHLH protein is not the cause of the observed phenotypes (Supplementary Figures 5B,C). Further support to this observation comes from previous studies where links between the strength of the *gun* phenotype and increments in mRNA level in *gun5* mutant as well as regulation of *CHLH*/*GUN5* gene by plastid signaling have been drawn (Moulin et al., 2008 and Supplementary Figure 7). It should be noted that clear differences in CHLH protein accumulation were found among the mutants with a similar *gun* phenotype, e.g., V679M and L690L (*rtl1*) (Figure 2A), but the CHLH protein levels significantly differed among the lines (Supplementary Figure 5C). Likewise, no significant relationship was observed for the CHLH protein level and the stomatal ABA phenotype (Figures 3, 4A,B, Supplementary Figures 4, 5A).

### Expression of truncated CHLH proteins (tCHLHs)

**Figure 5:**
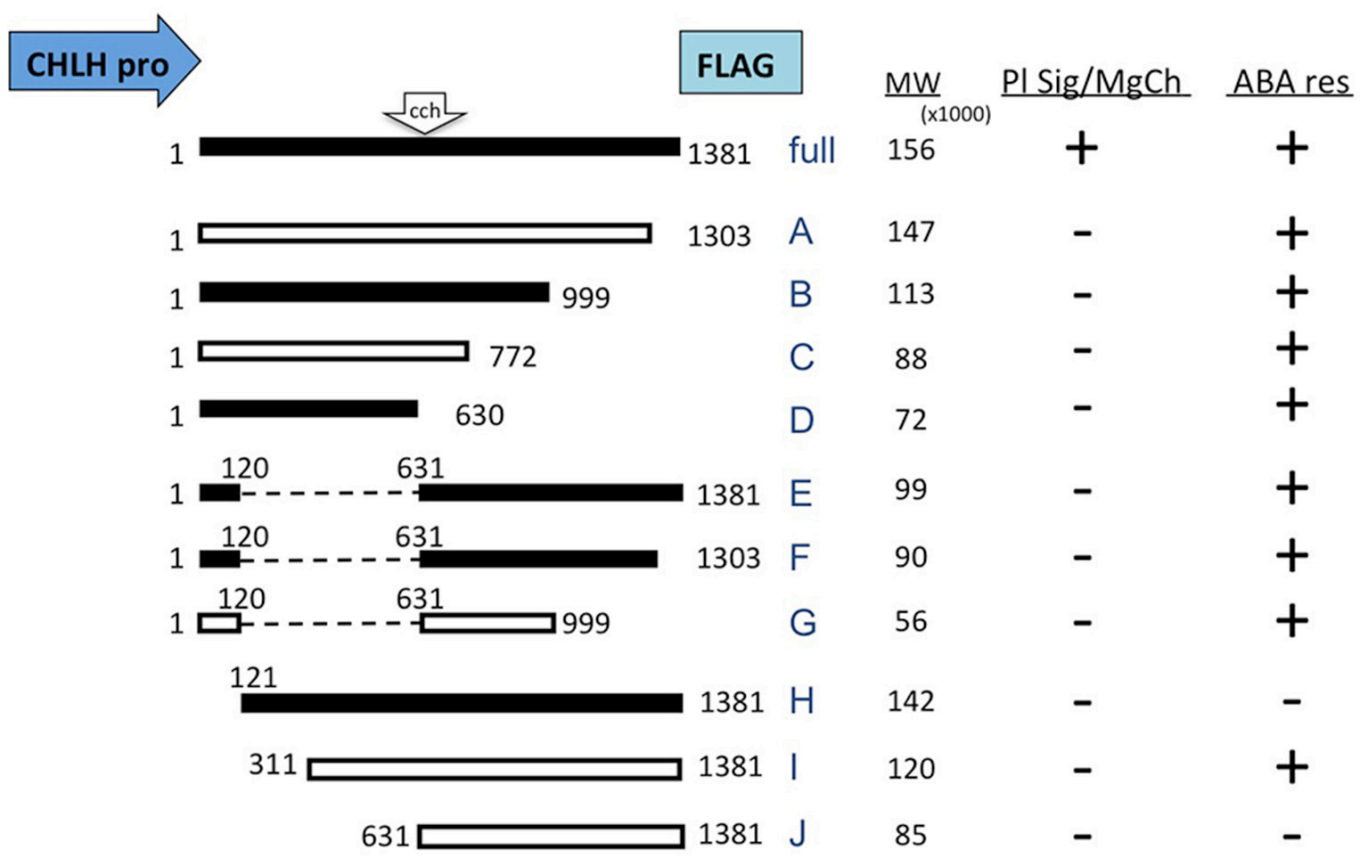
**Diagram of CHLH-full and truncated CHLH (tCHLH) constructs and a summary of phenotype analysis**. The constructs presented as white boxes contain the same fragments used in a previous work (Wu et al., 2009), and the constructs presented as black boxes were designed in this study. All the constructs carry the endogenous CHLH promoter (1.1 kb) and a C-terminal FLAG tag. The position of the *cch* mutation (P642L) is indicated at the top of the diagrams. The line IDs (full and A–J) and calculated molecular weights (parts per thousand) are indicated at the right of each construct. Internal deletions in tCHLH-E, -F, and -G are presented as dashed lines. A summary of phenotype assessments of plastid signaling and Mg-chelatase (Pl sig/MgCh) activity and ABA response of stomatal movement (ABA res) is presented at the right of the diagrams. “+”; complemented, “−”; not complemented.

We employed a different approach to gain insight into the domain structure of CHLH responsible for the individual physiological functions. Transgenic lines expressing truncated CHLH proteins have been used to delineate domains related to ABA responsiveness (Wu et al., 2009). Accordingly, we designed 11 different constructs with either full-length CHLH, a C-terminal, N-terminal, or internal deletion of CHLH, the sGFP-tag at the C-terminus and driven by the cauliflower mosaic virus 35S promoter (35Spro). These constructs were transformed into the *cch* mutant; however, most hygromycin-resistant T1 seedlings were albino and seedling lethal, which was most likely due to co-suppression or gene silencing. We thus replaced 35Spro with the endogenous promoter of *CHLH* and introduced FLAG as a C-terminal tag instead of sGFP (Figure 5). Utilizing these new constructs, we were able to obtain a reasonable number of transgenic plants, in which the expression of truncated CHLH (tCHLH) was tested by Western blotting using anti-CHLH and anti-FLAG antibodies. As shown in Supplementary Figures 8A–B, 9A–D, all lines expressed full-length GUN5-FLAG (CHLH-full) or tCHLH-FLAG at least during the seedling stage. Based on the band intensities using the anti-FLAG antibody (tCHLH-B as a reference), most tCHLH lines expressed an approximately 2-fold amount of the protein compared to wild type. Accumulation of tCHLH-H and tCHLH-J was especially low, and these lines were detected on overloaded blots in a separate experiment (Supplementary Figures 9C,D). Some lines exhibited reduced protein accumulation at the adult stage (Supplementary Figures 10A,B). We selected two or three representative lines among each tCHLH lines for the analysis below.

### Plastid signaling, Mg-chelatase and ABA phenotype in tCHLH lines

**Figure 6:**
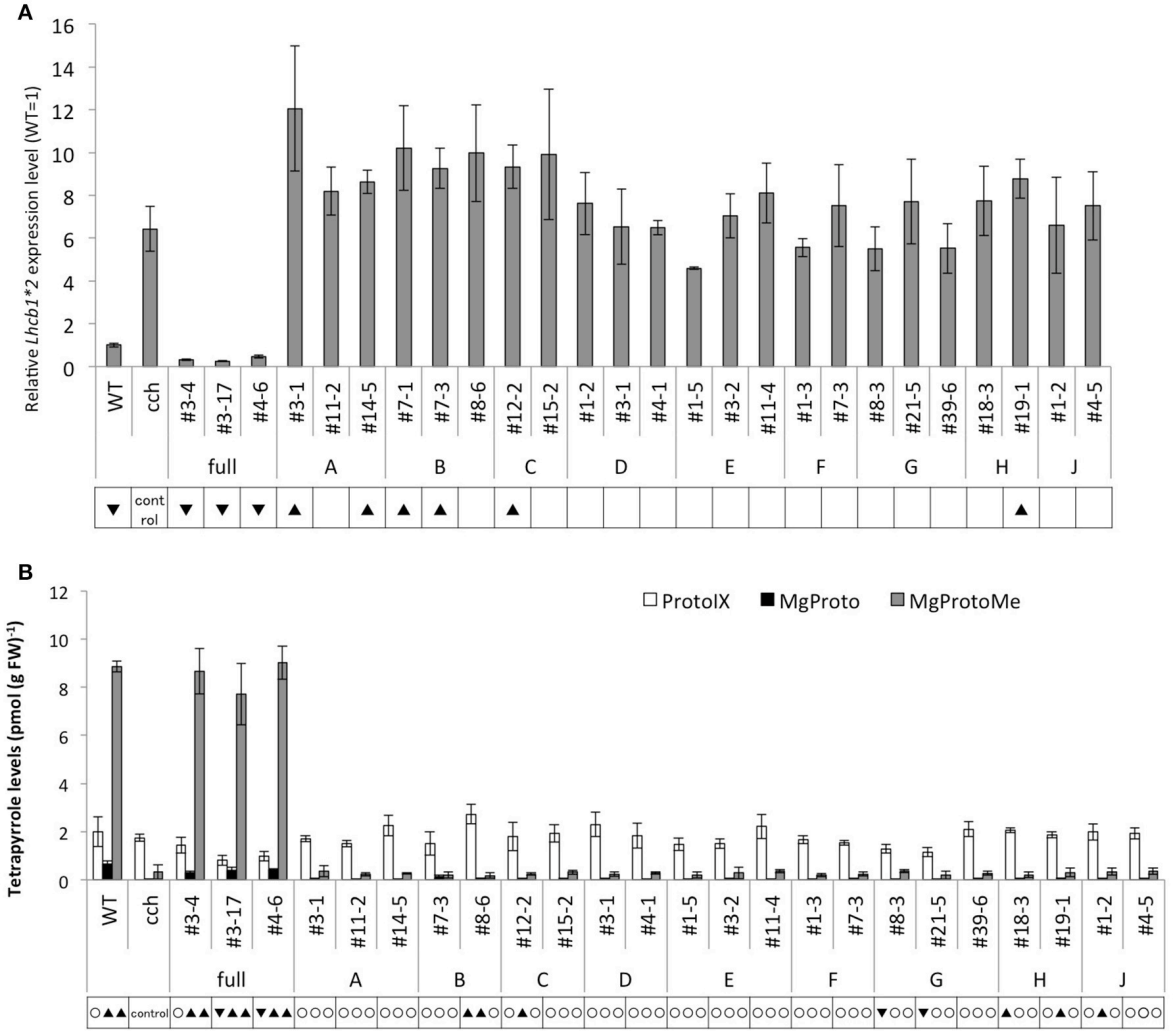
**Comparison of ***gun*** and tetrapyrrole phenotypes in wild type, ***cch***, and tCHLH-transgenic plants**. Plants were grown on MS medium supplemented with 2% sucrose and 2.5 μM NF for 5 days under continuous white light (100 μmol/m^2^/s). **(A)** *Lhcb1* mRNA levels in wild type and tCHLH plants grown in the presence of NF. *Lhcb1* mRNA levels were quantified and normalized to *TUB2* mRNA levels by RT-qPCR, as described in Materials and Methods. Data shown are the mean ± SD (*n* = 3), and the WT level is presented as 1.0. **(B)** Proto, MgProto, and MgProtoMe levels in wild type and tCHLH plants in the presence of NF. Tetrapyrrole was extracted and quantified by HPLC and normalized to fresh weight, as described in Materials and Methods. Proto (white bar), MgProto (black), and MgProtoMe (gray) levels are presented. Data shown are the mean ± SD (*n* ≧ 3). Statistical significance was determined by Student's *t*-test and the symbols are indicated as in Figure 2.

tCHLH lines were first examined for the *gun* phenotype in the presence of NF. The control line, CHLH-full, exhibited a wild type or even reduced Lhcb1^*^2 expression, complementing the *gun* phenotype (Figure 6A). In contrast, all other lines failed to complement and some showed enhanced *gun* phenotype (Figure 6A, Supplementary Figure 11A). CHLH-full did not significantly alter the gene expression levels of Lhcb1^*^2, while some of the tCHLH lines showed elevated expression of Lhcb1^*^2 in the absence of NF (Supplementary Figures 12C,D). Elevation of Lhcb1^*^2 expression in the absence or presence of NF observed in some tCHLH lines suggests dominant negative effect of these tCHLHs to *cch* mutation. Regarding the levels of tetrapyrroles (Proto, MgProto, and MgProtoMe), CHLH-full was able to complement *cch* in the absence or presence of NF (Figure 6B, Supplementary Figure 12A). On the other hand, the other tCHLH lines did not restore tetrapyrrole levels in the absence or presence of NF (Figure 6B, Supplementary Figures 11B, 12A,B), though statistically significant differences were detected in some lines when compared to the parental *cch* line. Likewise, chlorophyll-less phenotypes showed similar trends. CHLH-full restored chlorophyll content to wild type levels, while tCHLH lines showed equivalent or even lower abundance compared to the parental *cch* mutant (Supplementary Figures 11C,D). We were unable to reproduce the previous report that the expression of regions corresponding to tCHLH-A and tCHLH-G could complement both chlorophyll and tetrapyrrole phenotypes (ABARn and C370, respectively in Wu et al., 2009).

We then tested the tCHLH lines for sensitivity to ABA with respect to stomatal movement. Contrary to the results presented above, all tCHLH lines, except for tCHLH-H and tCHLH-J, restored the ABA sensitivity of guard cells, as shown in Supplementary Figure 13. The complementation failure observed in tCHLH-H and tCHLH-J may due to the lower abundance of tCHLH compared to other lines. In a previous study (Wu et al., 2009), the fragment containing a.a. 1–772 was unable to complement the *cch* stomatal phenotype, a result that led to the proposal that the C-terminal half, especially the region containing a.a. 631–999, is responsible for ABA sensitivity. However, we did observe recovery of ABA sensitivity in the tCHLH-B, -C, and -D lines, though the tCHLH-D does not overlap with the 631–999 region. These data suggest that the regions that complement the guard cell ABA-insensitive phenotype in the *cch* mutant is not limited to the C-terminal half of the CHLH protein but that the N-terminus also confers an equivalent ability (see the summary in Figure 5).

## Discussion

Different degrees of phenotype strength among *gun5* alleles have been reported, which suggests that specific regions (domains) of the CHLH protein correspond to functions of the Mg-chelatase subunit, plastid-to-nucleus signaling, and guard cell ABA responses (Mochizuki et al., 2001; Shen et al., 2006; Wu et al., 2009; Shang et al., 2010). To further understand the molecular nature of CHLH protein, we collected 12 newly identified and three previously characterized *gun5* missense mutant alleles and compared them with regard to phenotype. We also analyzed transgenic lines expressing a series of truncated CHLH proteins in *cch* mutant background. The summary of the analysis is shown in Figure 5 and Supplementary Figure 14.

### Analysis of plastid signaling, Mg-chelatase, and ABA response in *gun5* mutant alleles

In this study, we established that Proto and MgProtoMe levels in the presence of NF are positively and negatively correlated to the severity of *gun* phenotype in *gun5* mutants. Albeit the abundance of MgProto, a direct product of Mg-chelatase, is less correlated to the phenotype. The accumulation of Proto and reduction of MgProto(Me) can be explained by the attenuation of Mg-chelatase activity in *gun5* mutants. Moderate reduction of MgProto might involve a coordinated attenuation of both Mg-protoporphyrin IX methyltransferase and Mg-chelatase activities, although such co-regulation has not been reported. Taken together, these data support the hypotheses that: (a) MgProto(Me) functions as a negative plastid signal (Strand et al., 2003), and (b) that defect in the metabolic flow to the Mg-branch may increase the flow into the Fe-branch, and incremented heme level considered a positive plastid signal (Woodson et al., 2011; Terry and Smith, 2013). However, there are some exceptions. Mutations A1253V and R1290C at the C-terminus resulted in a normal level of tetrapyrrole intermediates with a weak but distinct *gun* phenotype. Although steady state levels of tetrapyrroles are not significantly changed in these mutants, they may have altered heme levels or a transient change in MgProto(Me) content as previously reported (Zhang et al., 2011). Future time-resolving profiling of tetrapyrroles including MgProto(Me) and heme may clarify our understanding the plastid signaling mechanism derived from tetrapyrrole metabolism and its intermediates.

Mutation in the central region of the CHLH protein, which corresponds to the C-terminal half of domains II and III in SynCHLH (Chen et al., 2015), has a strong impact on both Mg-chelatase activity and plastid signaling; nonetheless, as discussed below, regions important for CHLH function are also present around the N- and C-termini, such as residue S92, R1269, and R1290 (Figure 2, Supplementary Figure 14). Based on structural aspects of the CHLH protein, it has been proposed that domains III and V comprise a buried pocket porphyrin-binding structure (Chen et al., 2015). Hence, mutations in the central region may alter the affinity of CHLH for porphyrins, which results in the attenuation of Mg-chelatase activity and plastid signaling. However, one intriguing mutation, S92F, showed a strong *gun* phenotype comparable to that of mutations in the central region, though it has slight defects in tetrapyrrole levels. Because the predicted transit peptide region (a.a. 1–86) is likely to be removed upon entry into the plastid, S92 becomes situated near the N-terminus of CHLH (domain I in SynCHLH). According to the crystallographic analysis of SynCHLH, domains I and V form a dimerization interface; accordingly, S92F might interfere with the interaction between CHLH molecules (Supplementary Figure 14). Although an enhancing effect of the N-terminal fragment was previously reported using a reconstructed *Synechocystis* Mg-chelatase reaction (Sirijovski et al., 2008), the present study reports the first indication of the importance of the CHLH N-terminus to Mg-chelatase activity and plastid signaling *in vivo*. The residues at C-terminal region, A1253, R1269, and R1290, appear to be important for plastid signaling though they have less contribution to Mg-chelatase activity. Because the domains containing these residues (domain I & the end of V to VI) locate outer region of the CHLH protein, the mutation in these residues may interfere with the interaction of other components such as CHLI, CHLD, and GUN4 that are involved in plastid signaling (Strand et al., 2003; Huang and Li, 2009; Adhikari et al., 2011).

Contrary to the chlorophyll and plastid signaling phenotypes, mutations affecting the stomatal ABA response had no clear relationship with these two phenotypes. For instance, G286E, R464C, and T984I are newly identified mutations with no ABA phenotype, even though they result in a relatively strong *gun* phenotype (Figure 2A, Supplementary Figures 4, 14A). Conversely, A1253V exhibits defects in ABA response but causes a weak *gun* phenotype. Among the six alleles that showed stomatal phenotypes, P462L (*cch*) and L690F (*rtl1*) are located in the region corresponding to the ABA-binding core (Wu et al., 2009), whereas the remaining four mutations are located in different regions of the CHLH protein, regions that have not previously been suggested to be involved in ABA signaling. We identified two novel mutations (P450L and A1253V) that showed constitutively closed stomatal phenotype and appeared to have defect in the signal transduction pathway leading to the activation of H^+^-ATPase (Figure 4). A similar stomatal phenotype was observed when the CHLH-GFP fusion protein was overexpressed in guard cells under the epidermis-specific *CER6* promoter (Tsuzuki et al., 2013). However, this does not appear to be the case for P450L and A1253V because we did not detect a significant increase in CHLH protein levels in these mutants (Supplementary Figure 5A) and genetic analysis of these mutants clearly showed that the mutations are both recessive (Figures 4A,B). For one intriguing mutation, V679M, the substitution of Val with Met results in a perfect match with the SynCHLH sequence (see Supplementary Figure 1). However, this “SynCHLH”-type substitution causes AtCHLH deficiency in plastid signaling and Mg-chelatase activity (Figures 2A,B, Supplementary Figures 2A,B). As V679 (M632 in SynCHLH) is located close to the residues that form the internal pocket, the internal pocket structure might be greatly affected by this substitution, even though the substitution is “conserved.”

We were unable to identify *gun5* mutants for domain IV, and only one mutation (A1253V) at the peripheral region of domain V was found. Mutation in this region may cause severe defects in Mg-chelatase activity and therefore be seedling lethal. The analysis of such difficult regions may be facilitated using inducible deactivation of CHLH activity as described previously (Schlicke et al., 2014).

### Analysis of plastid signaling, Mg-chelatase, and ABA response in tCHLH lines

As an additional experiment, we adopted a gain-of-function approach by expressing truncated CHLH (tCHLH) in the *cch* mutant background and assessed the three physiological functions. Transgenic lines expressing full-length CHLH (CHLH-full) completely complemented the *cch* mutation in all three phenotypes (Figure 6, Supplementary Figures 11C, 12A). All other tCHLH lines were unable to complement the phenotypes. Some lines even showed dominant negative effects on the chlorophyll and *gun* phenotype, suggesting tCHLH interference of CHLH protein function in *cch* mutant. Contrary to our results, it was previously reported that a.a. 1–1303 and 1–120 + 631–999 (corresponding to tCHLH-A and tCHLH-G, respectively) can complement the chlorophyll phenotype of *cch* (Wu et al., 2009). This difference in results may be due to the relatively lower expression level of tCHLHs compared to that in the previous report. Contrary to the chlorophyll and plastid signaling phenotypes, the C-terminal (tCHLH-A to -D) and the middle+C-terminal deletion lines (tCHLH-E, -F, and -G), and an N-terminal deleted line (tCHLH-I) recovered guard cell sensitivity to ABA (Supplementary Figure 13). It should be noted that both tCHLH-C and tCHLH-D could rescue the ABA phenotype of *cch*, even though they do not contain the region (a.a. 631–999) proposed to play a central role in ABA signaling (Wu et al., 2009). This finding suggests that either the N-terminal or C-terminal half of CHLH is sufficient for ABA response in guard cells or that these regions might interact with the *cch* (P642L) mutant form of the CHLH protein, partially restoring function. The latter idea is somewhat supported by the report that the N-terminal half of SynCHLH enhances Mg-chelatase activity *in vitro*, though such an enhancing effect of the C-terminal half has not been reported (Sirijovski et al., 2008). It is intriguing that C-terminal half (tCHLH-I), which lacks the authentic plastid transit peptide, can complement the ABA phenotype of *cch*. A similar result was reported previously, whereby truncated CHLH consisting of a.a. 311–1381 or 631–1381 (corresponding to tCHLH-I and tCHLH-J) can complement the *cch* mutation (Wu et al., 2009). These authors showed that GFP-tagged fragments localize to the cytoplasm, and according to their model, the CHLH protein is embedded in the plastid envelope and the N- and C-terminal regions are exposed on the cytosolic surface of the plastid. Accordingly, C-terminal region interacts with cytosolic components such as WRKY transcription factors, SOAR1 and SnRK2.6/OST1 to induce ABA-responses.

It should be noted that the effect of truncated CHLHs has only been tested in the wild type or *cch* mutant background (Wu et al., 2009; Shang et al., 2010; this study). Therefore, the alterations caused by tCHLHs might be dependent on the presence of wild type or *cch*-form CHLH proteins. We were unable to address this point in view of tCHLHs lack of complementation of the seedling lethal phenotype of a T-DNA knockout mutant. Thereafter, structural domains analyses should be carefully performed in combination with other methodologies such as random mutagenesis of the CHLH protein and inducible deactivation of CHLH activity (Schlicke et al., 2014).

## Author contributions

NM designed the research. AN, HI, and NM performed research. HI and NM analyzed data and wrote the article.

### Conflict of interest statement

The authors declare that the research was conducted in the absence of any commercial or financial relationships that could be construed as a potential conflict of interest.

## Supplementary material

Supplementary Figure 1 **Amino acid sequence alignment of ***Synechocystis*** and ***Arabidopsis*** CHLHs**. Amino acid sequences of *Synechocystis* (BAA17040.1) and *Arabidopsis* (CAA92802.1) CHLH proteins were aligned using CLUSTALW. The domain structures based on crystallography analysis of SynCHLH are indicated below the sequences respectively as (I) blue, (II) green, (III) orange, (IV) brown, (V) magenta, and (VI) purple lines (Chen et al., 2015). The amino acid positions that are substituted in *gun5* mutants are indicated by triangles with the allele names.

Supplementary Figure 2 **Comparison of chlorophyll, tetrapyrrole intermediates and ***Lhcb1***^*****^***2*** mRNA levels in wild type (WT) and ***gun5*** mutants grown in the absence of NF. (A)** Chlorophyll accumulation. Plants were grown on MS medium supplemented with 2% sucrose for 5 days under continuous white light (100 μmol/m^2^/s). Chlorophyll a is presented as a dark-gray bar and chlorophyll b as a light-gray bar, respectively. Data shown are the mean ± SD (*n* = 3). **(B)** Proto, MgProto, and MgProtoMe levels. Plants were grown as described in **(A)**. Tetrapyrrole was extracted and quantified by HPLC and normalized to fresh weight, as described in Materials and Methods. Proto (white bar), MgProto (black), and MgProtoMe (gray) levels are presented. Data shown are the mean ± SD (*n* ≧ 3). **(C)** *Lhcb1* mRNA levels. Plants were grown as described in **(A)**. *Lhcb1* mRNA levels were quantified and normalized to *TUB2* mRNA levels by RT- qPCR, as described in Materials and Methods. Data shown are the mean ± SD (*n* = 3), and the WT level is presented as 1.0. Statistical significance was determined by Student's *t*-test and the symbols are indicated as in Figure 2 in the main text.

Supplementary Figure 3 **Correlation between ***Lhcb1***^*****^***2*** mRNA and tetrapyrrole levels**. Tetrapyrrole intermediate and chlorophyll levels are plotted against the *Lhcb1* mRNA levels, and Pearson's correlation coefficient (*r*) and significance probability (*p*) are presented in the graph. **(A–C)** Proto, MgProto, and MgProtoMe levels in −NF condition vs. *Lhcb1* mRNA levels in +NF condition. **(D–F)** Proto, MgProto, and MgProtoMe levels in +NF condition vs. *Lhcb1* mRNA levels in +NF condition. **(G,H)** Chlorophyll vs. Lhcb1 mRNA levels in −NF **(G)** or in +NF **(H)**, respectively.

Supplementary Figure 4 **Inhibition of light-induced stomatal opening in ***gun5*** mutants by ABA**. Effect of ABA on light-induced stomatal opening in *gun5* mutants showing normal ABA sensitivity. Experiments were performed as described in Figure 3 and Materials and Methods. The light and ABA treatment regimes were as follows: red light at 50 μmol/m^2^/s in the absence (red bar) or presence (white bar) of 20 μM ABA (+ABA); blue light at 10 μmol/m^2^/s with background red light at 50 μmol/m^2^/s in the absence (blue) or presence (gray) of 20 μM ABA. Statistical significance was determined by Student's *t*-test. ^*^*p* < 0.05.

Supplementary Figure 5 **Comparison of CHLH protein levels in ***gun5*** mutants. (A)** The CHLH protein levels of 4-week-old wild type and *gun5* mutant plants grown in soil under long-day condition. CHLH proteins were detected using an anti-CHLH antibody. Total protein (10 μg) was applied to each lane. The relative protein levels are indicated above the lanes (WT as 1.0). **(B,C)** CHLH protein levels in 5-day-old wild type and *gun5* mutant seedlings grown on MS medium in the absence **(B)** or presence **(C)** of 2.5 μM NF. Total protein (20 μg) was applied to each lane. The relative protein levels are indicated below the lanes (WT, control as 1.0). The blot image was spliced and re-aligned for visualization.

Supplementary Figure 6 **Inhibition of seed germination and growth in ***gun5*** mutants by ABA**. Wild type and *gun5* mutant seeds were sown on MS agar medium containing 2% (w/v) sucrose in the absence **(A)** or presence **(B)** of 0.5 μM ABA, and the plates were incubated for 8 days after stratification as described in Materials and Methods. The scale bar is 5 mm.

Supplementary Figure 7 *****CHLH*** mRNA levels in wild type and ***gun5*** mutant alleles**. CHLH mRNA levels in wild type and *gun5* mutants grown in the absence **(A)** or presence **(B)** of NF. *CHLH* mRNA levels were quantified and normalized to *TUB2* mRNA levels by RT-qPCR, as described in Materials and Methods. Data shown are the mean ± SD (*n* = 3), and the WT level is presented as 1.0. Statistical significance was determined by Student's *t*-test and the symbols are indicated as in Figure 2 in the main text.

Supplementary Figure 8 **Comparison of CHLH protein levels in tCHLH lines (tCHLH-A, -B, -C, -D) in 5-day-old seedlings. (A,B)** CHLH protein levels in 5-day-old wild type, *cch* and tCHLH-A, -B, -C, -D seedlings grown on MS medium in the absence **(A)** or presence **(B)** of 2.5 μM NF. Total protein (20 μg) was applied to each lane. Proteins were first detected using the anti-CHLH antibody (top), followed by the anti-FLAG antibody (bottom) after stripping the first antibody. RBCL bands on the stained membrane are indicated as loading controls in **(A)**. For **(B)**, RBCL bands are unclear due to the NF treatment, thus anonymous bands are indicated as loading controls. The position of the endogenous CHLH protein is indicated by an asterisk.

Supplementary Figure 9 **Comparison of CHLH protein levels in tCHLH lines (tCHLH-E, -F, -G, -H, -I) in 5-day-old seedlings. (A,B)** CHLH protein levels in 5-day-old wild type, *cch* and tCHLH-E, -F, -G, -H, -I seedlings grown on MS medium in the absence **(A)** or presence **(B)** of 2.5 μM NF. Total protein (20 μg) was applied to each lane. Proteins were first detected using the anti-CHLH antibody (top), followed by the anti-FLAG antibody (bottom) after stripping the first antibody. The position of the endogenous CHLH protein is indicated by an asterisk. The blot image was spliced and re-aligned for visualization. **(C,D)** CHLH protein levels of 5-day-old wild type, *cch* and tCHLH-H, -I, -J seedlings grown on MS medium in the absence **(C)** or presence **(D)** of 2.5 μM NF. To enhance detection, approximately 60 μg total protein was applied to each lane and detected using the anti-FLAG antibody. Total protein extract (20 μg) of tCHLH-D #4−1 was used as a positive control. In **(A,C)**, RBCL bands on the stained membrane are indicated as loading controls. For **(B,C)**, anonymous bands are indicated as loading controls as described in Supplementary Figure 8.

Supplementary Figure 10 **Comparison of CHLH protein levels in tCHLH lines in 4-week-old plants. (A,B)** CHLH protein levels in 4-week-old wild type, *cch* and tCHLH plants grown in soil under long-day conditions. Total protein (20 μg) was applied to each lane. Proteins were first detected using the anti-CHLH antibody (top), followed by the anti-FLAG antibody (bottom) after stripping the first antibody. The position of the endogenous CHLH protein is indicated by an asterisk. The blot image was spliced and re-aligned for visualization. RBCL bands on the stained membrane are indicated as loading controls.

Supplementary Figure 11 **Comparison of ***gun*** and chlorophyll phenotypes in wild type and tCHLH lines. (A,B,D)** Characterization of the tCHLH-I line was performed separately from other tCHLH lines due to the limited availability of seeds. **(A)** *Lhcb1* mRNA levels in wild type, *cch* and tCHLH-I plants in the presence of NF. *Lhcb1* mRNA levels were quantified and normalized to *TUB2* mRNA levels by RT-qPCR, as described in Materials and Methods. Data shown are the mean ± SD (*n* = 3), and the WT level is presented as 1.0. **(B)** Proto, MgProto, and MgProtoMe levels in wild type, *cch* and tCHLH-I plants in the presence of NF. Tetrapyrrole was extracted and quantified by HPLC and normalized to fresh weight, as described in Materials and Methods. Proto (white bar), MgProto (black), and MgProtoMe (gray) levels are presented. Data shown are the mean ± SD (*n* ≧ 3). **(C,D)** Chlorophyll accumulation. Plants were grown on MS medium supplemented with 2% sucrose for 5 days under continuous white light (100 μmol/m^2^/s). Chlorophyll a is presented as a dark-gray bar and chlorophyll b as a light-gray bar, respectively. Data shown are the mean values for **(C)** (*n* = 2), and mean ± SD for **(D)** (*n* = 3). Statistical significance was determined by Student's *t*-test and the symbols are indicated as in Figure 2 in the main text.

Supplementary Figure 12 **Comparison of ***gun*** and tetrapyrrole phenotypes in wild type and tCHLH plants grown in the absence of NF. (A,B)** Proto, MgProto, and MgProtoMe levels in wild type, *cch* and tCHLH plants in the absence of NF. **(B)** Tetrapyrrole levels in tCHLH-I were assessed separately from other lines due to the limited availability of seeds. Data shown are the mean ± SD (*n* ≧ 3). **(C,D)** *Lhcb1* mRNA levels in wild type, *cch*, CHLH-full and tCHLH plants in the absence of NF. *Lhcb1* mRNA levels were quantified and normalized to *TUB2* mRNA levels by RT-qPCR. (D) *Lhcb1* mRNA levels in tCHLH-I were assessed separately from other lines due to the limited availability of seeds. Data shown are the mean ± SD (*n* = 3), and the WT level is presented as 1.0. **(D)** There is no significant difference in the average value between the wild type and tCHLH-I lines. Statistical significance was determined by Student's *t*-test and the symbols are indicated as in Figure 2 in the main text.

Supplementary Figure 13 **Inhibition of light-induced stomatal opening in tCHLH lines by ABA**. Effect of ABA on light-induced stomatal opening in tCHLH lines. The experiments were performed as described in Figure 3. Light and ABA treatment regimes are as follows: red light at 50 μmol/m^2^/s in the absence (red bar); blue light at 10 μmol/m^2^/s with background red light at 50 μmol/m^2^/s in the absence (blue) or presence (gray) of 20 μM ABA. Statistical significance was determined by Student's *t*-test. ^*^*p* < 0.05.

Supplementary Figure 14 **Schematic diagrams of the currently proposed functional and structural domain architecture of the CHLH protein, and the positions of the amino acid substitutions and phenotypes in the *gun5* mutants used in this study**. (Top) Black and gray boxes represent the CHLH protein and the transit peptide, respectively. Wild type amino acids and positions and mutant amino acids are indicated. **(A)** Vertical black and gray lines represent mutants showing strong or moderate phenotypes in the assays indicated in the left, respectively. Asterisks indicate the mutants exhibiting constitutively closed stomatal phenotypes. **(B)** Green boxes represent the proposed domain structures based on crystallography analysis of SynCHLH (Chen et al., 2015). **(C)** White boxes represent the proposed regions responsible for ABA binding, the transmembrane domain and the WRKY interaction domain (Wu et al., 2009; Shang et al., 2010).

Supplementary Table 1 **Primers used in this study**.
