# Supplementary figures and images for: CHLH/GUN5 Function in Tetrapyrrole Metabolism Is Correlated with Plastid Signaling but not ABA Responses in Guard Cells
Source: Front Plant Sci. 2016 Nov 7;7:1650. doi: 10.3389/fpls.2016.01650 (PMC5098175; doi:10.3389/fpls.2016.01650)

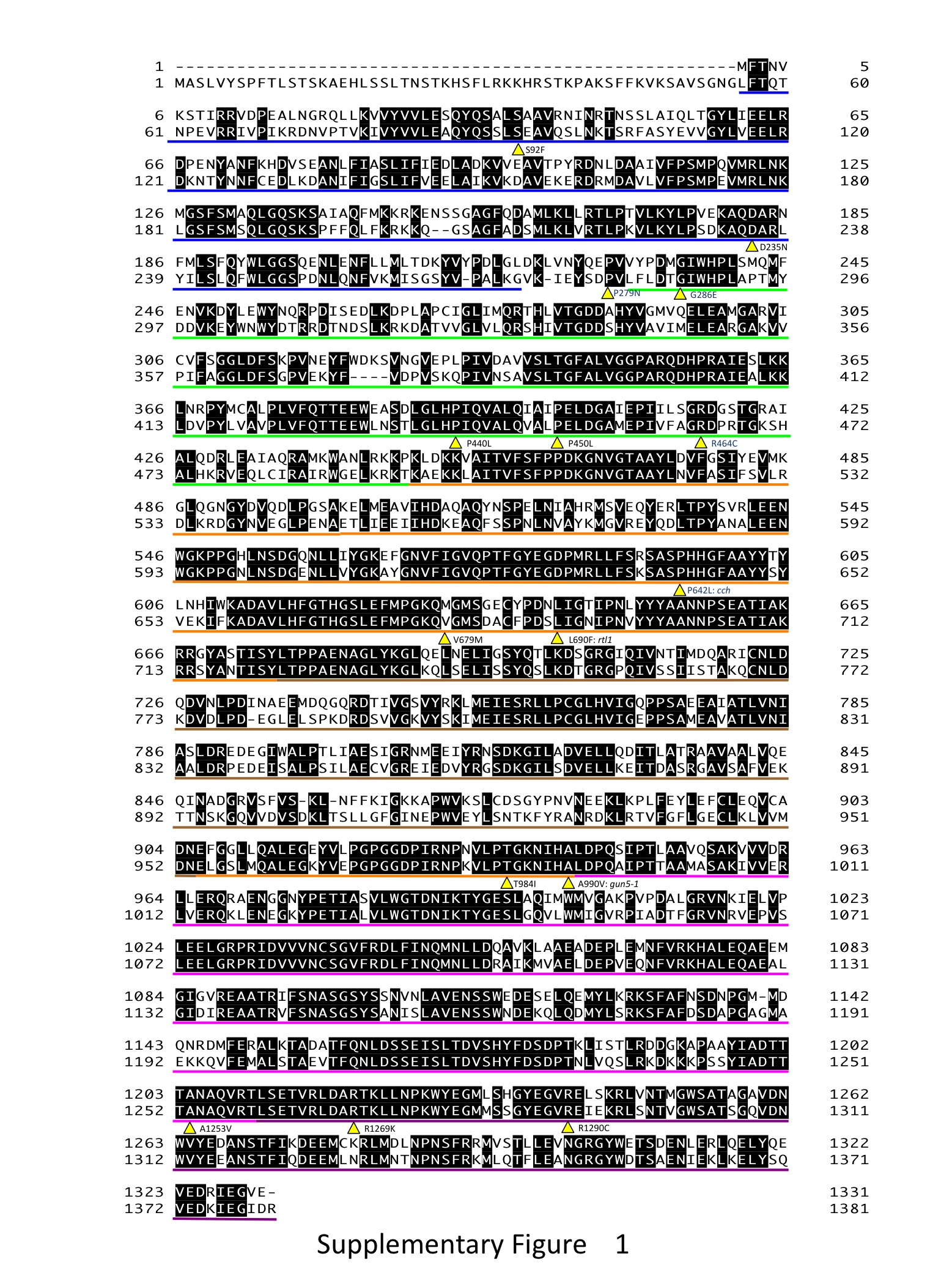

Supplement: Supplementary Figure 1 — Amino acid sequence alignment of Synechocystis and Arabidopsis CHLHs. Amino acid sequences of Synechocystis (BAA17040.1) and Arabidopsis (CAA92802.1) CHLH proteins were aligned using CLUSTALW. The domain structures based on crystallography analysis of SynCHLH are indicated below the sequences respectively as (I) blue, (II) green, (III) orange, (IV) brown, (V) magenta, and (VI) purple lines (Chen et al., 2015). The amino acid positions that are substituted in gun5 mutants are indicated by triangles with the allele names. [file Image1.JPEG]

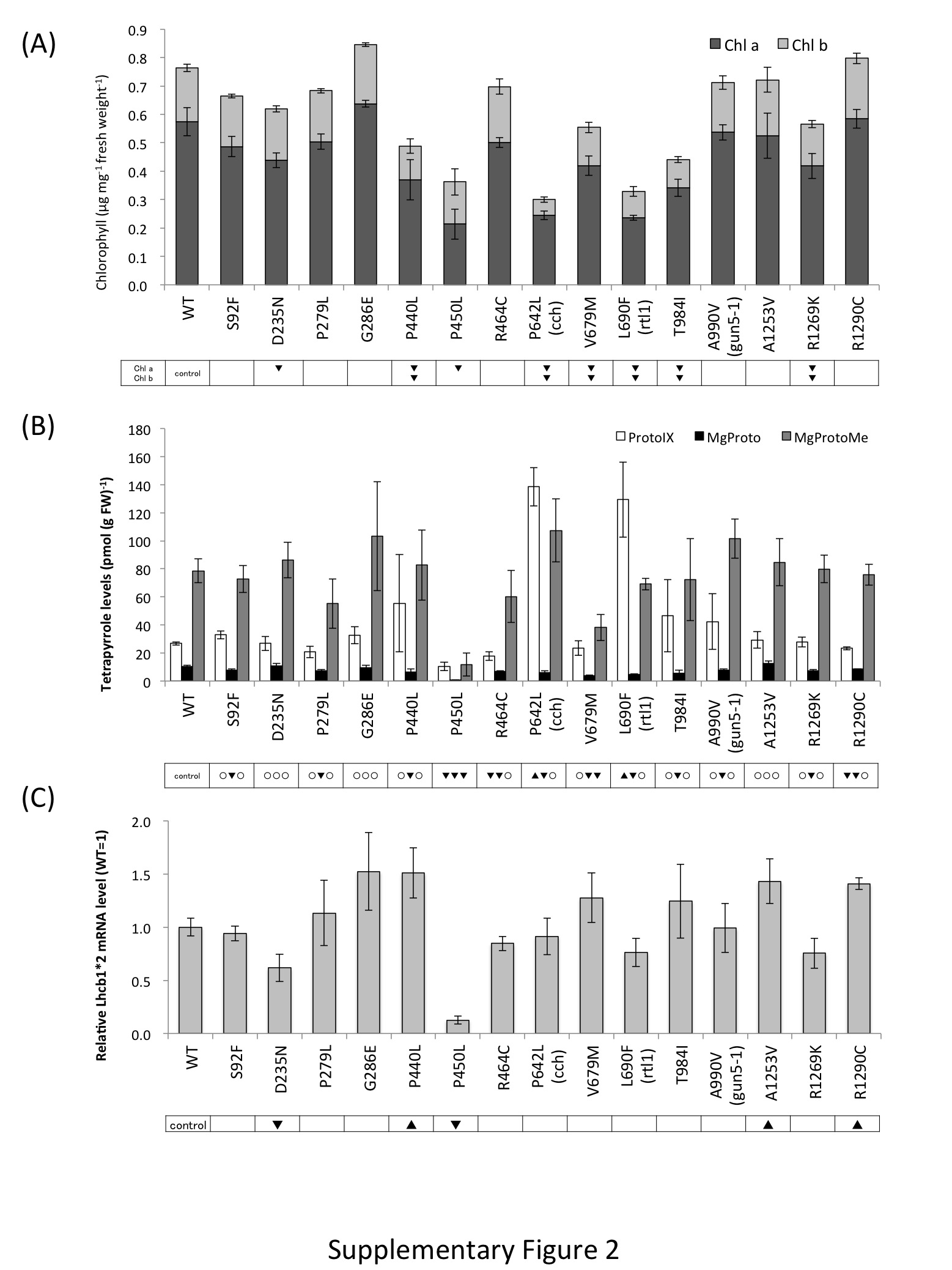

Supplement: Supplementary Figure 2 — Comparison of chlorophyll, tetrapyrrole intermediates and Lhcb1*2 mRNA levels in wild type (WT) and gun5 mutants grown in the absence of NF. (A) Chlorophyll accumulation. Plants were grown on MS medium supplemented with 2% sucrose for 5 days under continuous white light (100 μmol/m2/s). Chlorophyll a is presented as a dark-gray bar and chlorophyll b as a light-gray bar, respectively. Data shown are the mean ± SD (n = 3). (B) Proto, MgProto, and MgProtoMe levels. Plants were grown as described in (A). Tetrapyrrole was extracted and quantified by HPLC and normalized to fresh weight, as described in Materials and Methods. Proto (white bar), MgProto (black), and MgProtoMe (gray) levels are presented. Data shown are the mean ± SD (n ≧ 3). (C) Lhcb1 mRNA levels. Plants were grown as described in (A). Lhcb1 mRNA levels were quantified and normalized to TUB2 mRNA levels by RT- qPCR, as described in Materials and Methods. Data shown are the mean ± SD (n = 3), and the WT level is presented as 1.0. Statistical significance was determined by Student's t-test and the symbols are indicated as in Figure 2 in the main text. [file Image2.JPEG]

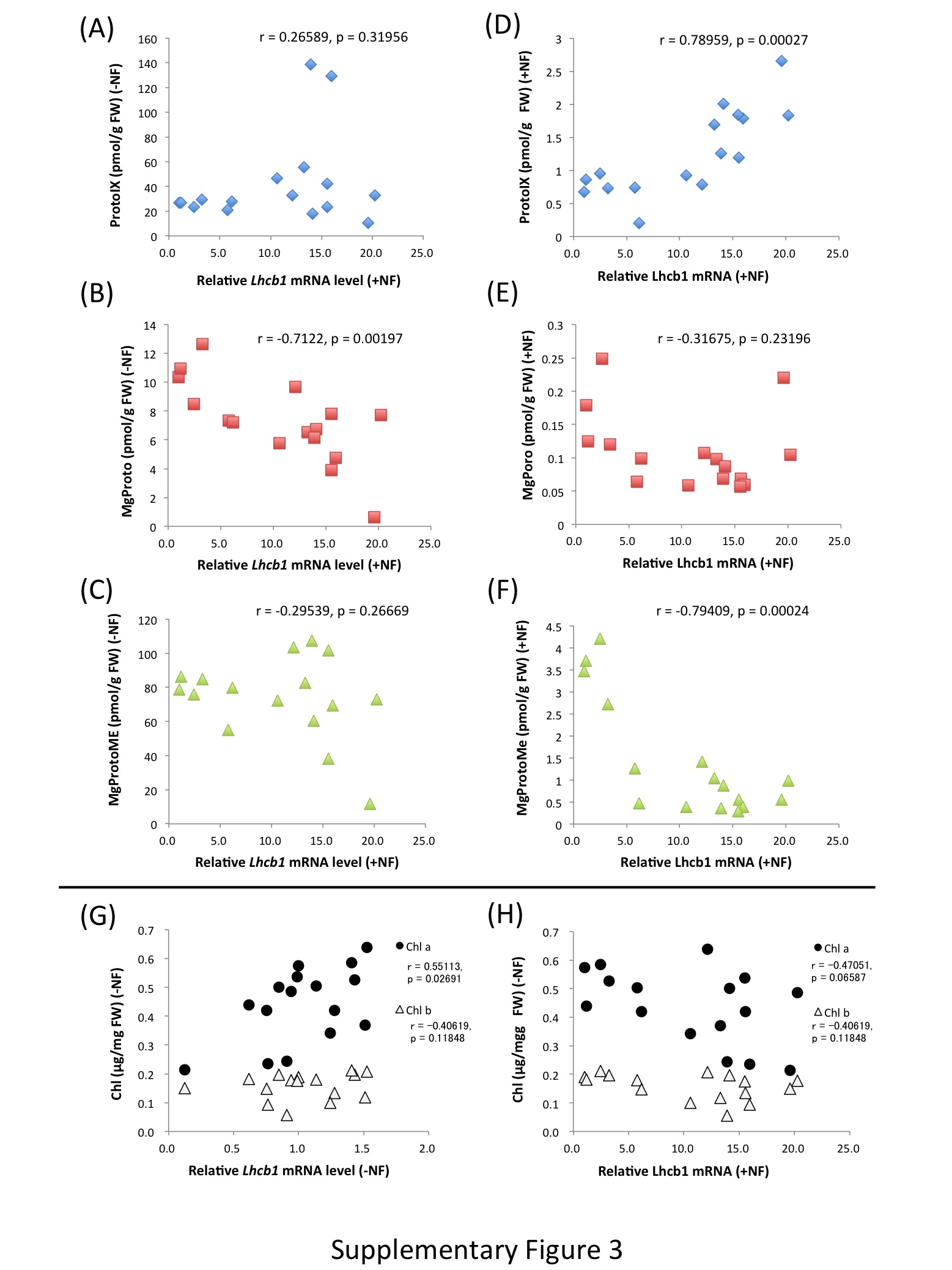

Supplement: Supplementary Figure 3 — Correlation between Lhcb1*2 mRNA and tetrapyrrole levels. Tetrapyrrole intermediate and chlorophyll levels are plotted against the Lhcb1 mRNA levels, and Pearson's correlation coefficient (r) and significance probability (p) are presented in the graph. (A–C) Proto, MgProto, and MgProtoMe levels in −NF condition vs. Lhcb1 mRNA levels in +NF condition. (D–F) Proto, MgProto, and MgProtoMe levels in +NF condition vs. Lhcb1 mRNA levels in +NF condition. (G,H) Chlorophyll vs. Lhcb1 mRNA levels in −NF (G) or in +NF (H), respectively. [file Image3.JPEG]

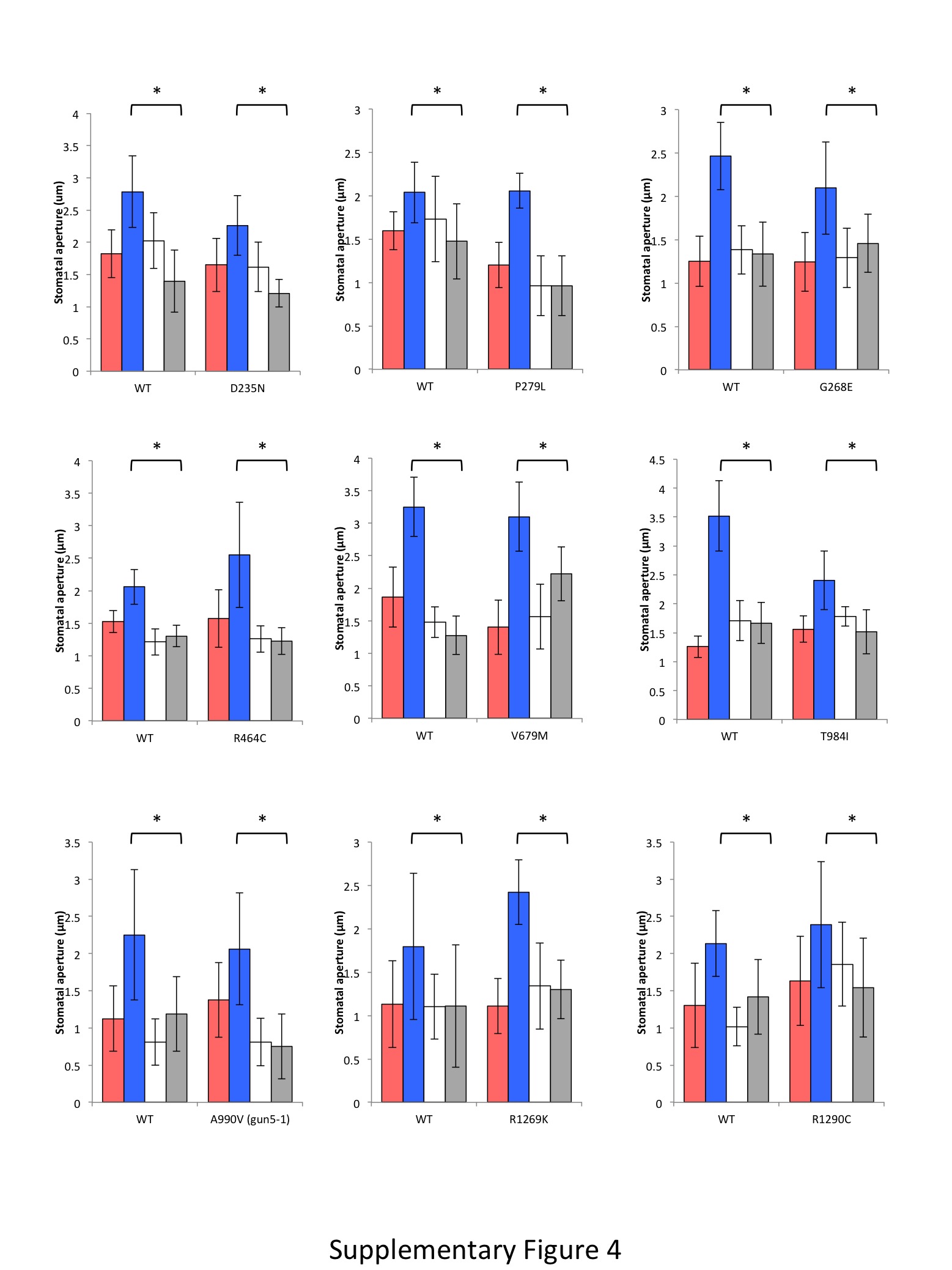

Supplement: Supplementary Figure 4 — Inhibition of light-induced stomatal opening in gun5 mutants by ABA. Effect of ABA on light-induced stomatal opening in gun5 mutants showing normal ABA sensitivity. Experiments were performed as described in Figure 3 and Materials and Methods. The light and ABA treatment regimes were as follows: red light at 50 μmol/m2/s in the absence (red bar) or presence (white bar) of 20 μM ABA (+ABA); blue light at 10 μmol/m2/s with background red light at 50 μmol/m2/s in the absence (blue) or presence (gray) of 20 μM ABA. Statistical significance was determined by Student's t-test. *p < 0.05. [file Image4.JPEG]

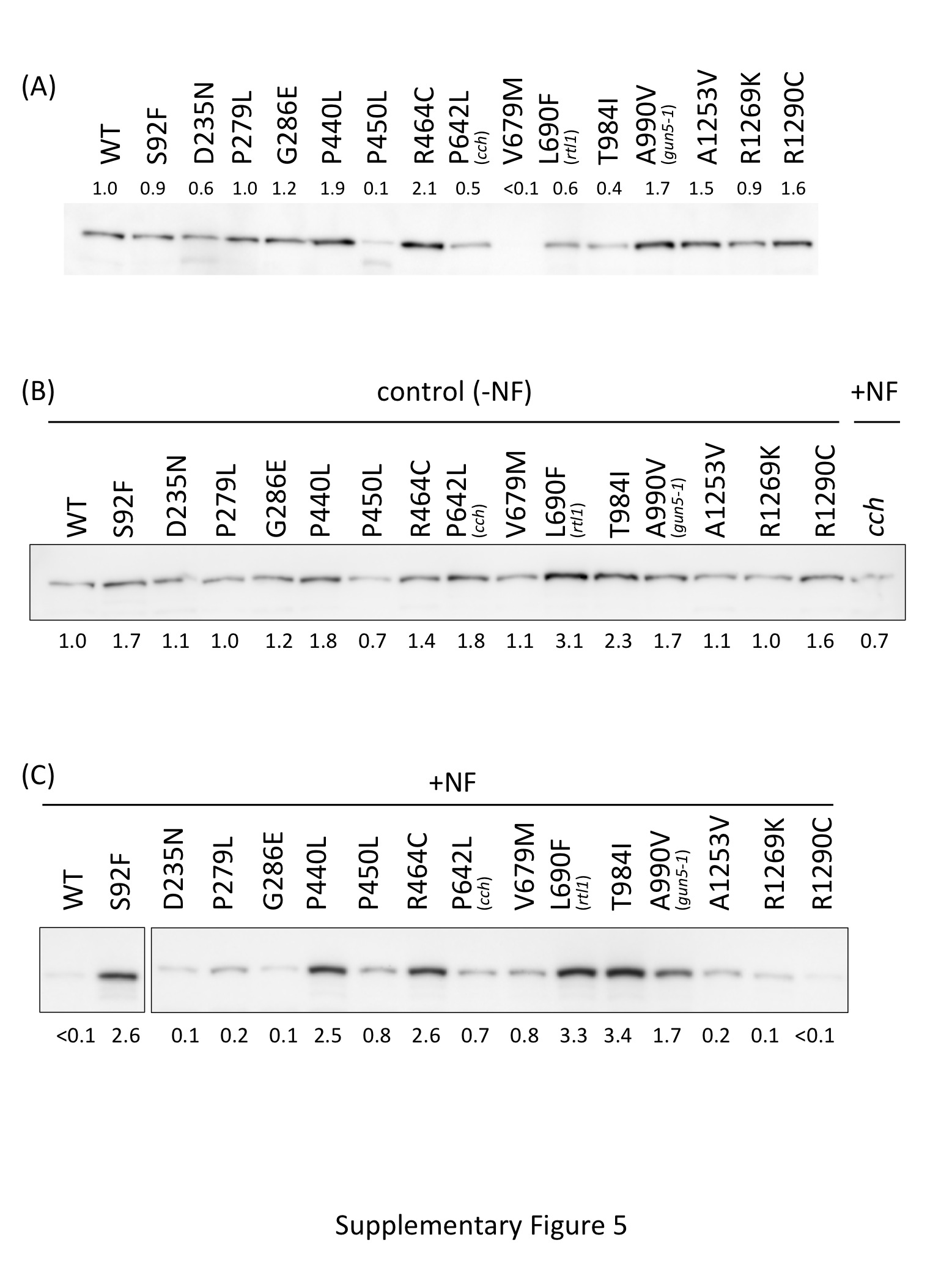

Supplement: Supplementary Figure 5 — Comparison of CHLH protein levels in gun5 mutants. (A) The CHLH protein levels of 4-week-old wild type and gun5 mutant plants grown in soil under long-day condition. CHLH proteins were detected using an anti-CHLH antibody. Total protein (10 μg) was applied to each lane. The relative protein levels are indicated above the lanes (WT as 1.0). (B,C) CHLH protein levels in 5-day-old wild type and gun5 mutant seedlings grown on MS medium in the absence (B) or presence (C) of 2.5 μM NF. Total protein (20 μg) was applied to each lane. The relative protein levels are indicated below the lanes (WT, control as 1.0). The blot image was spliced and re-aligned for visualization. [file Image5.JPEG]

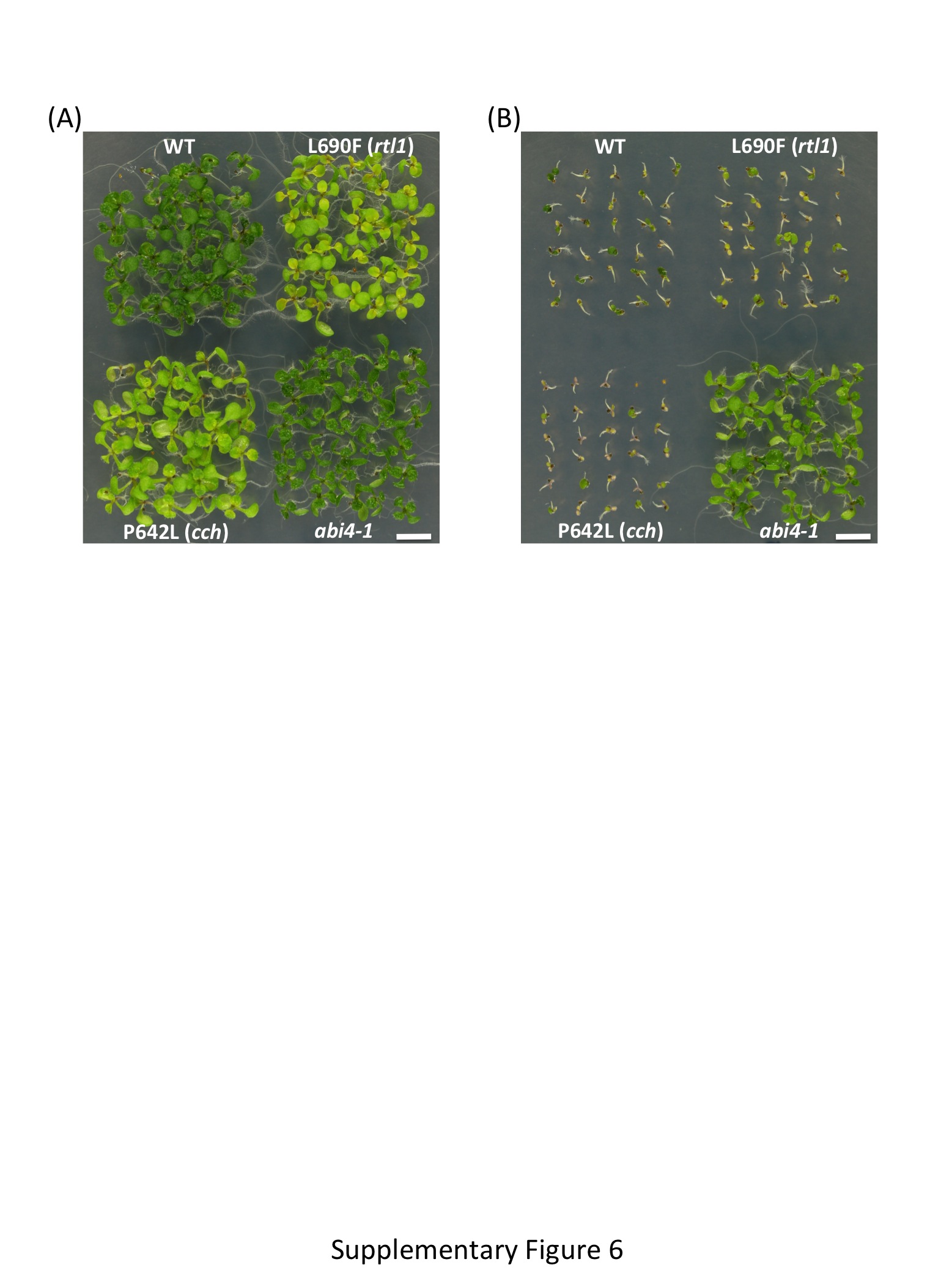

Supplement: Supplementary Figure 6 — Inhibition of seed germination and growth in gun5 mutants by ABA. Wild type and gun5 mutant seeds were sown on MS agar medium containing 2% (w/v) sucrose in the absence (A) or presence (B) of 0.5 μM ABA, and the plates were incubated for 8 days after stratification as described in Materials and Methods. The scale bar is 5 mm. [file Image6.JPEG]

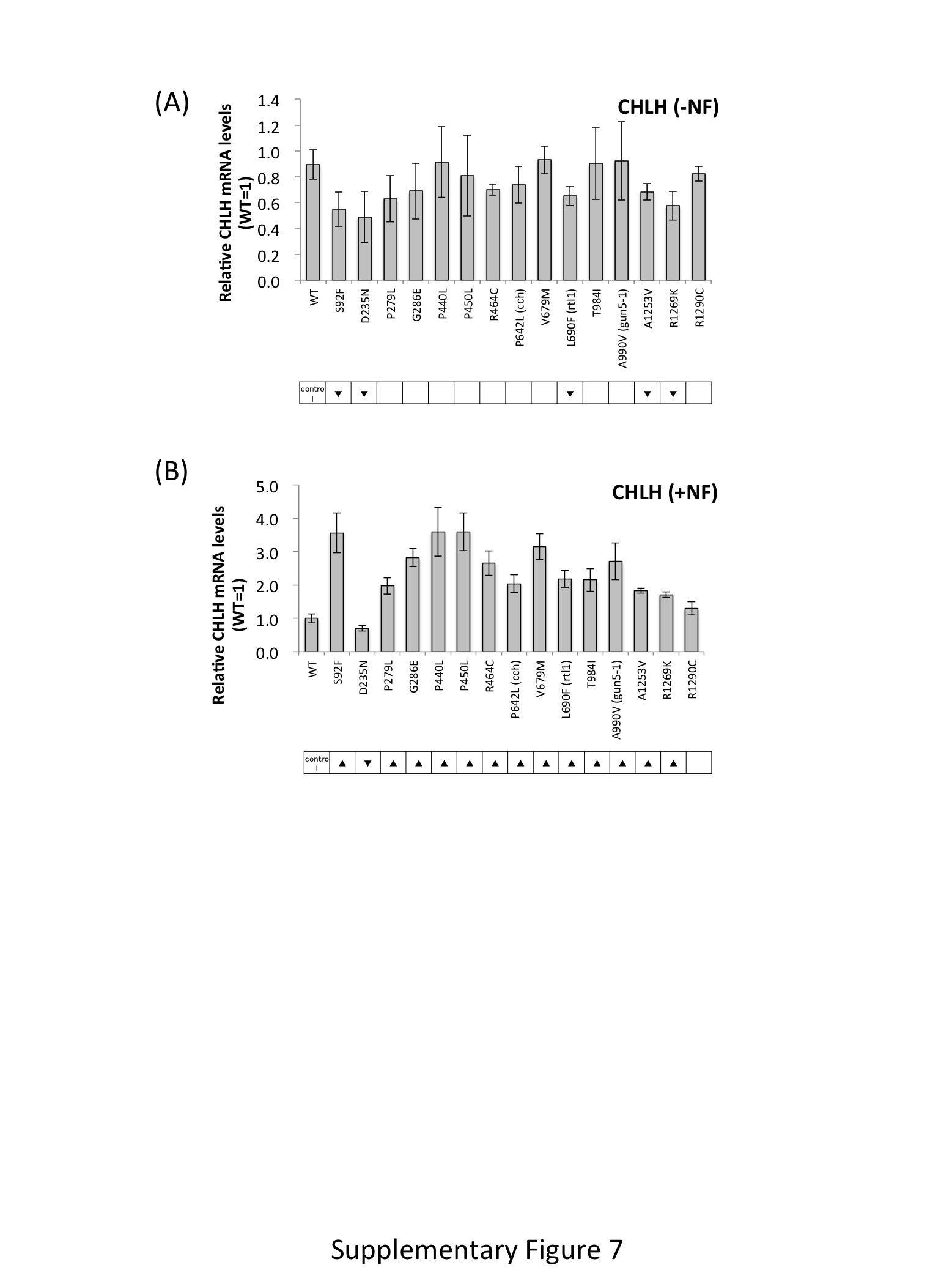

Supplement: Supplementary Figure 7 — CHLH mRNA levels in wild type and gun5 mutant alleles. CHLH mRNA levels in wild type and gun5 mutants grown in the absence (A) or presence (B) of NF. CHLH mRNA levels were quantified and normalized to TUB2 mRNA levels by RT-qPCR, as described in Materials and Methods. Data shown are the mean ± SD (n = 3), and the WT level is presented as 1.0. Statistical significance was determined by Student's t-test and the symbols are indicated as in Figure 2 in the main text. [file Image7.JPEG]

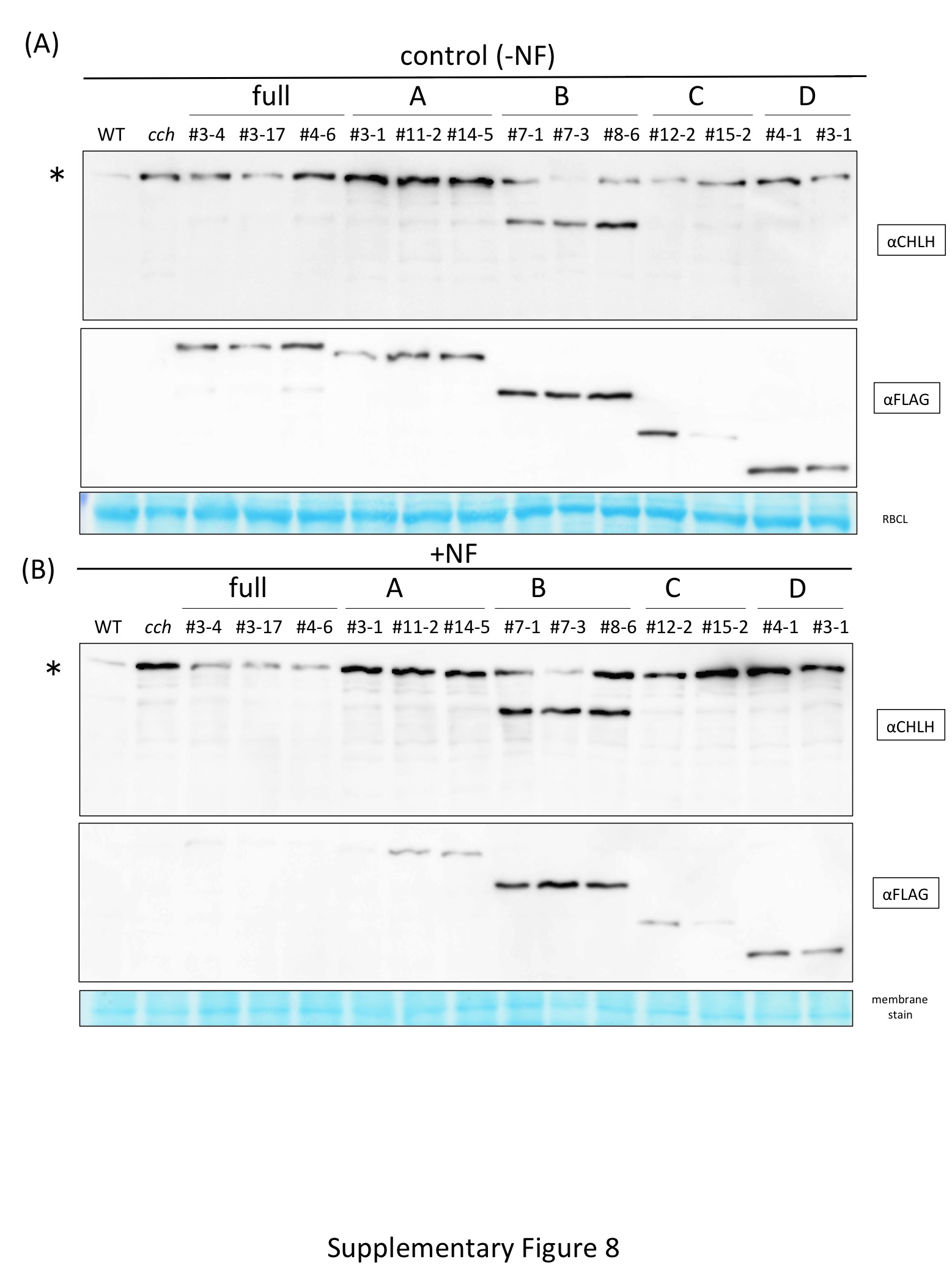

Supplement: Supplementary Figure 8 — Comparison of CHLH protein levels in tCHLH lines (tCHLH-A, -B, -C, -D) in 5-day-old seedlings. (A,B) CHLH protein levels in 5-day-old wild type, cch and tCHLH-A, -B, -C, -D seedlings grown on MS medium in the absence (A) or presence (B) of 2.5 μM NF. Total protein (20 μg) was applied to each lane. Proteins were first detected using the anti-CHLH antibody (top), followed by the anti-FLAG antibody (bottom) after stripping the first antibody. RBCL bands on the stained membrane are indicated as loading controls in (A). For (B), RBCL bands are unclear due to the NF treatment, thus anonymous bands are indicated as loading controls. The position of the endogenous CHLH protein is indicated by an asterisk. [file Image8.JPEG]

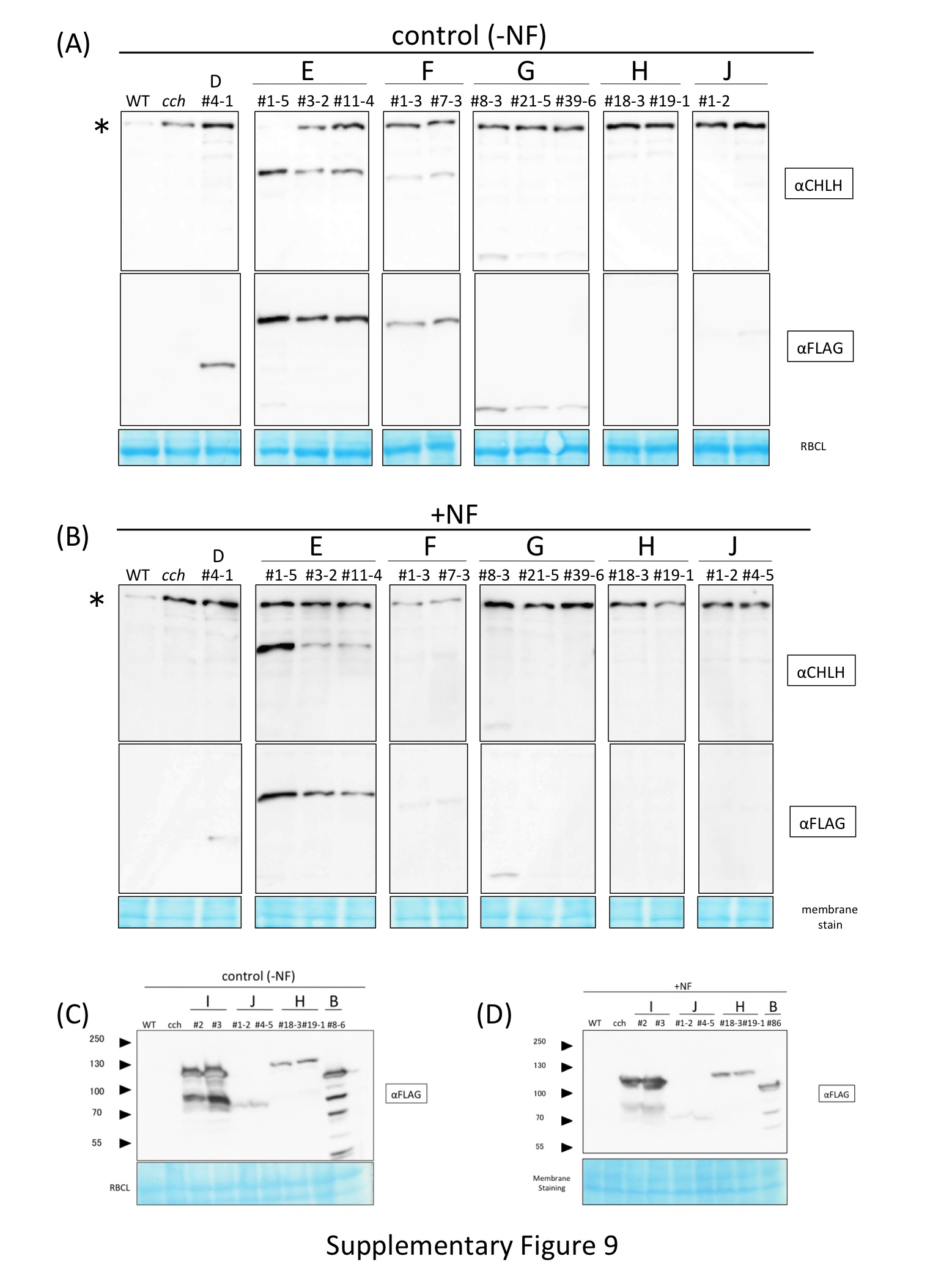

Supplement: Supplementary Figure 9 — Comparison of CHLH protein levels in tCHLH lines (tCHLH-E, -F, -G, -H, -I) in 5-day-old seedlings. (A,B) CHLH protein levels in 5-day-old wild type, cch and tCHLH-E, -F, -G, -H, -I seedlings grown on MS medium in the absence (A) or presence (B) of 2.5 μM NF. Total protein (20 μg) was applied to each lane. Proteins were first detected using the anti-CHLH antibody (top), followed by the anti-FLAG antibody (bottom) after stripping the first antibody. The position of the endogenous CHLH protein is indicated by an asterisk. The blot image was spliced and re-aligned for visualization. (C,D) CHLH protein levels of 5-day-old wild type, cch and tCHLH-H, -I, -J seedlings grown on MS medium in the absence (C) or presence (D) of 2.5 μM NF. To enhance detection, approximately 60 μg total protein was applied to each lane and detected using the anti-FLAG antibody. Total protein extract (20 μg) of tCHLH-D #4−1 was used as a positive control. In (A,C), RBCL bands on the stained membrane are indicated as loading controls. For (B,C), anonymous bands are indicated as loading controls as described in Supplementary Figure 8. [file Image9.JPEG]

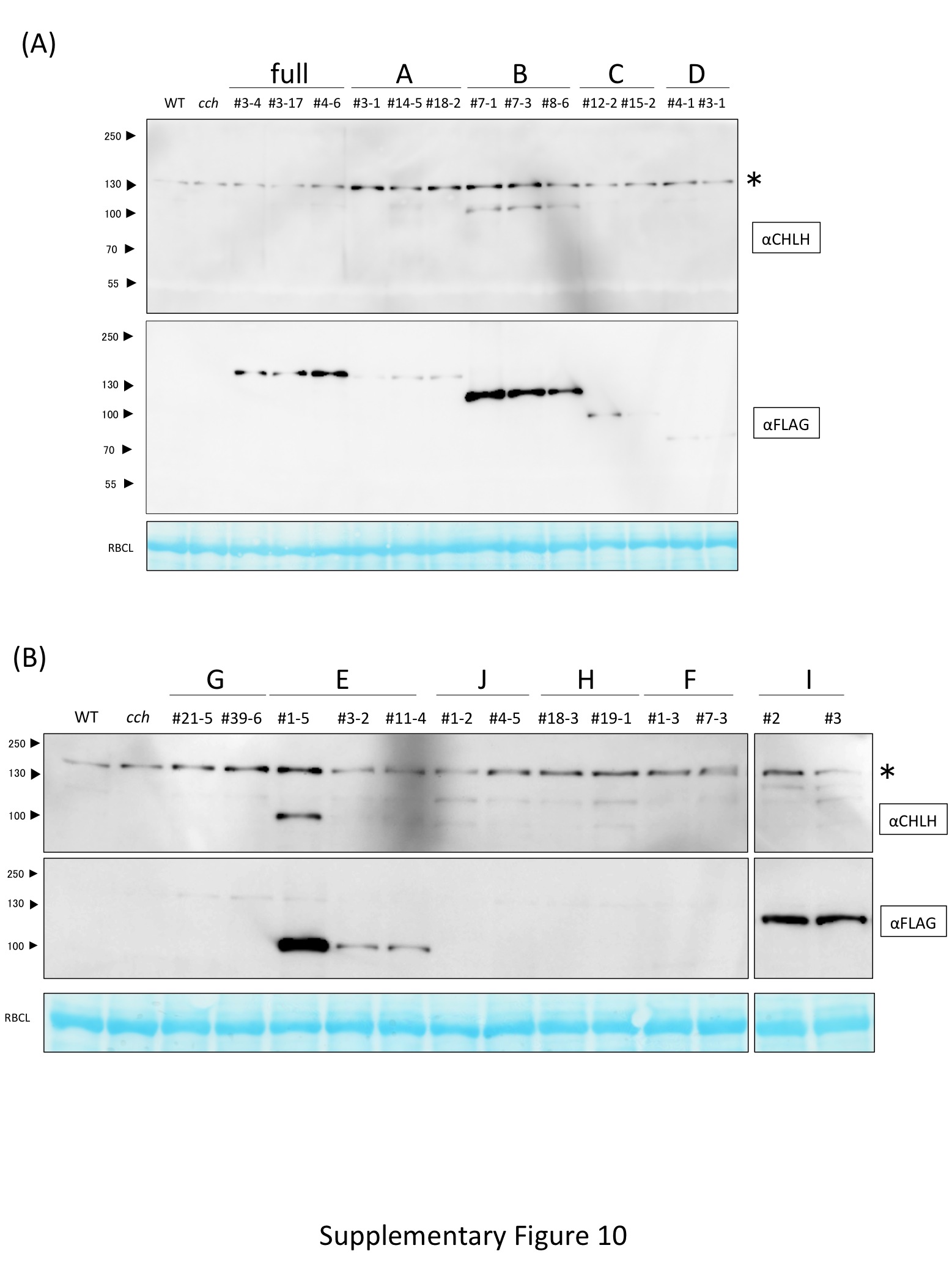

Supplement: Supplementary Figure 10 — Comparison of CHLH protein levels in tCHLH lines in 4-week-old plants. (A,B) CHLH protein levels in 4-week-old wild type, cch and tCHLH plants grown in soil under long-day conditions. Total protein (20 μg) was applied to each lane. Proteins were first detected using the anti-CHLH antibody (top), followed by the anti-FLAG antibody (bottom) after stripping the first antibody. The position of the endogenous CHLH protein is indicated by an asterisk. The blot image was spliced and re-aligned for visualization. RBCL bands on the stained membrane are indicated as loading controls. [file Image10.JPEG]

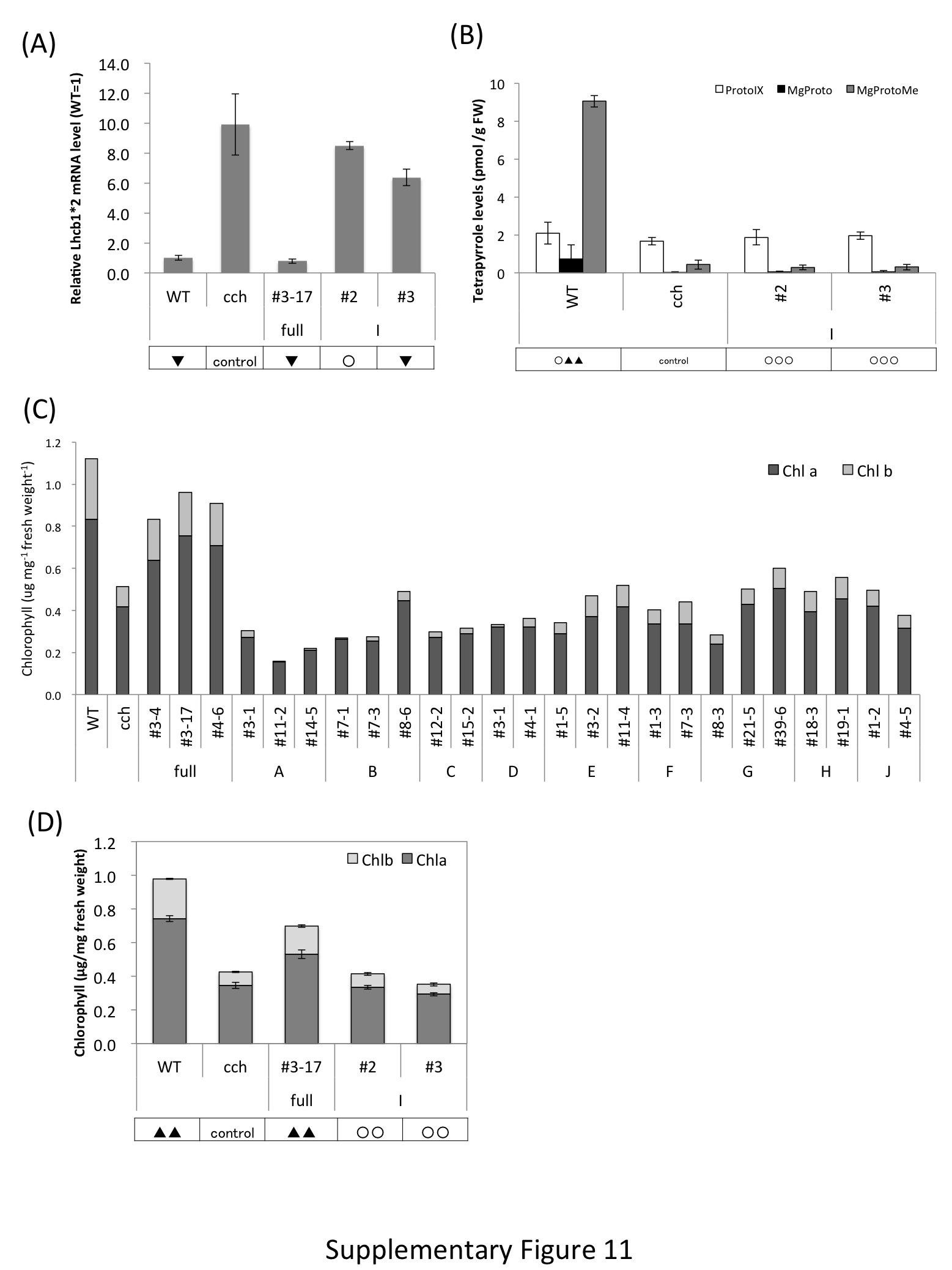

Supplement: Supplementary Figure 11 — Comparison of gun and chlorophyll phenotypes in wild type and tCHLH lines. (A,B,D) Characterization of the tCHLH-I line was performed separately from other tCHLH lines due to the limited availability of seeds. (A) Lhcb1 mRNA levels in wild type, cch and tCHLH-I plants in the presence of NF. Lhcb1 mRNA levels were quantified and normalized to TUB2 mRNA levels by RT-qPCR, as described in Materials and Methods. Data shown are the mean ± SD (n = 3), and the WT level is presented as 1.0. (B) Proto, MgProto, and MgProtoMe levels in wild type, cch and tCHLH-I plants in the presence of NF. Tetrapyrrole was extracted and quantified by HPLC and normalized to fresh weight, as described in Materials and Methods. Proto (white bar), MgProto (black), and MgProtoMe (gray) levels are presented. Data shown are the mean ± SD (n ≧ 3). (C,D) Chlorophyll accumulation. Plants were grown on MS medium supplemented with 2% sucrose for 5 days under continuous white light (100 μmol/m2/s). Chlorophyll a is presented as a dark-gray bar and chlorophyll b as a light-gray bar, respectively. Data shown are the mean values for (C) (n = 2), and mean ± SD for (D) (n = 3). Statistical significance was determined by Student's t-test and the symbols are indicated as in Figure 2 in the main text. [file Image11.JPEG]

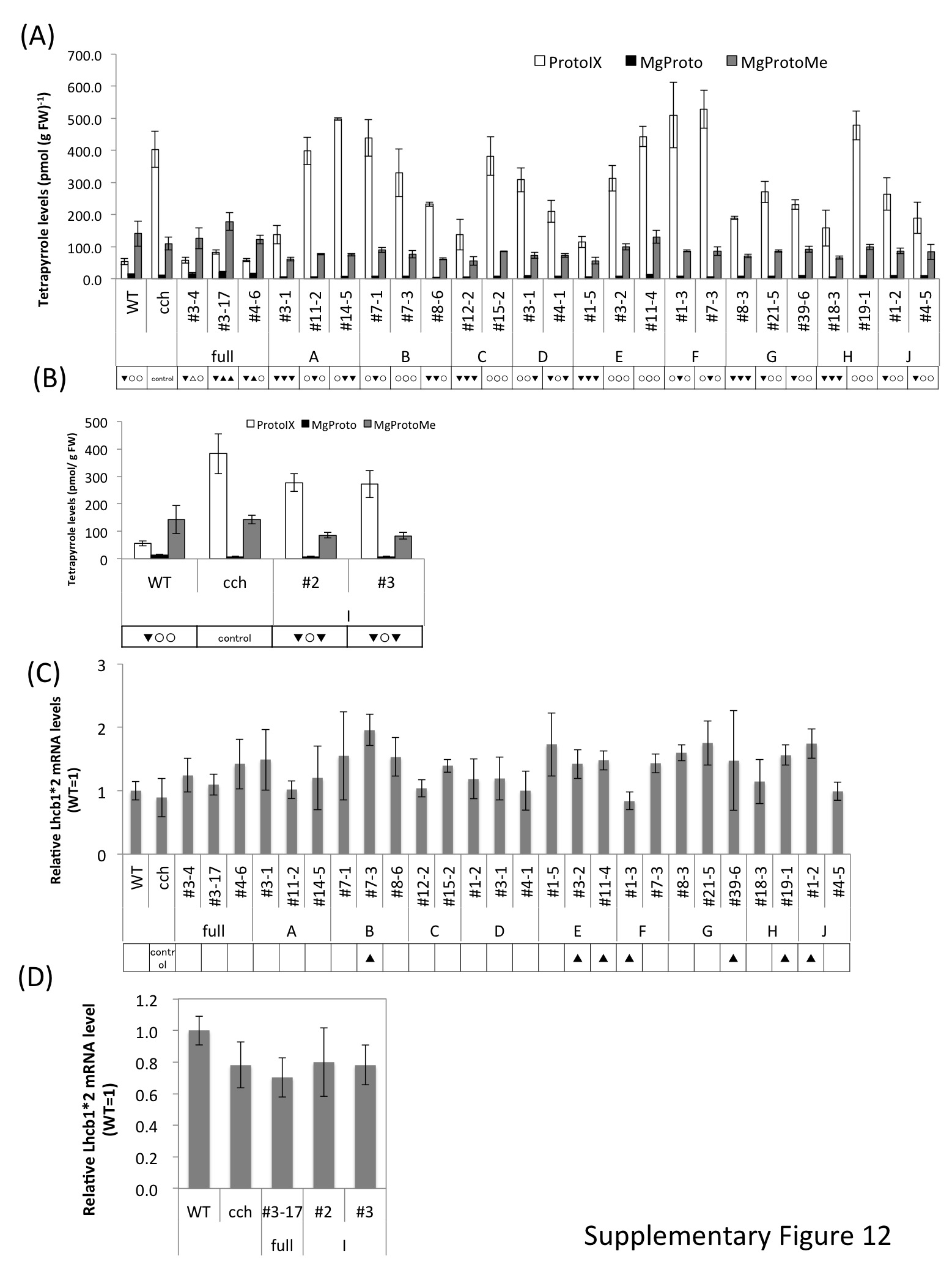

Supplement: Supplementary Figure 12 — Comparison of gun and tetrapyrrole phenotypes in wild type and tCHLH plants grown in the absence of NF. (A,B) Proto, MgProto, and MgProtoMe levels in wild type, cch and tCHLH plants in the absence of NF. (B) Tetrapyrrole levels in tCHLH-I were assessed separately from other lines due to the limited availability of seeds. Data shown are the mean ± SD (n ≧ 3). (C,D) Lhcb1 mRNA levels in wild type, cch, CHLH-full and tCHLH plants in the absence of NF. Lhcb1 mRNA levels were quantified and normalized to TUB2 mRNA levels by RT-qPCR. (D) Lhcb1 mRNA levels in tCHLH-I were assessed separately from other lines due to the limited availability of seeds. Data shown are the mean ± SD (n = 3), and the WT level is presented as 1.0. (D) There is no significant difference in the average value between the wild type and tCHLH-I lines. Statistical significance was determined by Student's t-test and the symbols are indicated as in Figure 2 in the main text. [file Image12.JPEG]

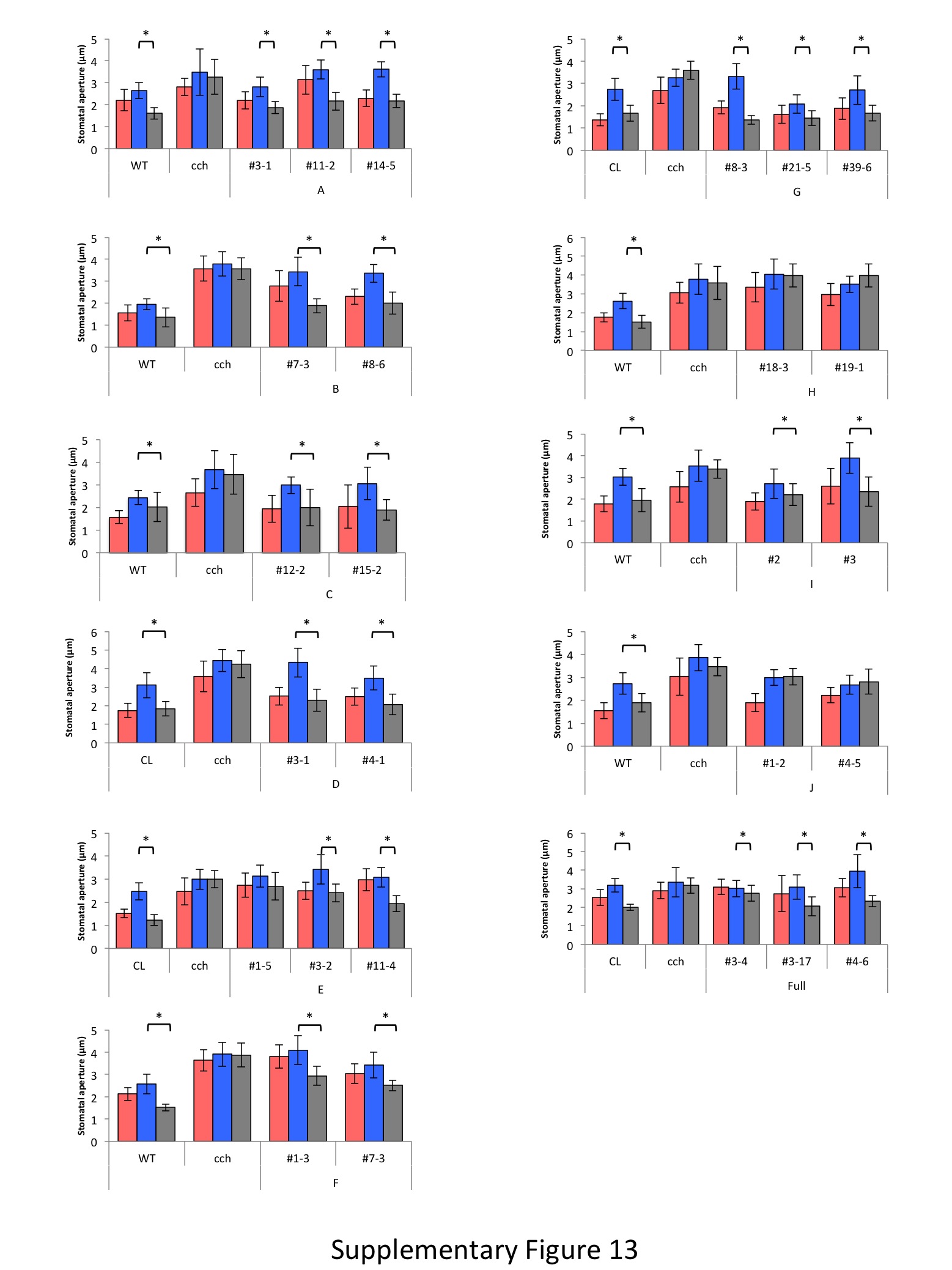

Supplement: Supplementary Figure 13 — Inhibition of light-induced stomatal opening in tCHLH lines by ABA. Effect of ABA on light-induced stomatal opening in tCHLH lines. The experiments were performed as described in Figure 3. Light and ABA treatment regimes are as follows: red light at 50 μmol/m2/s in the absence (red bar); blue light at 10 μmol/m2/s with background red light at 50 μmol/m2/s in the absence (blue) or presence (gray) of 20 μM ABA. Statistical significance was determined by Student's t-test. *p < 0.05. [file Image13.JPEG]

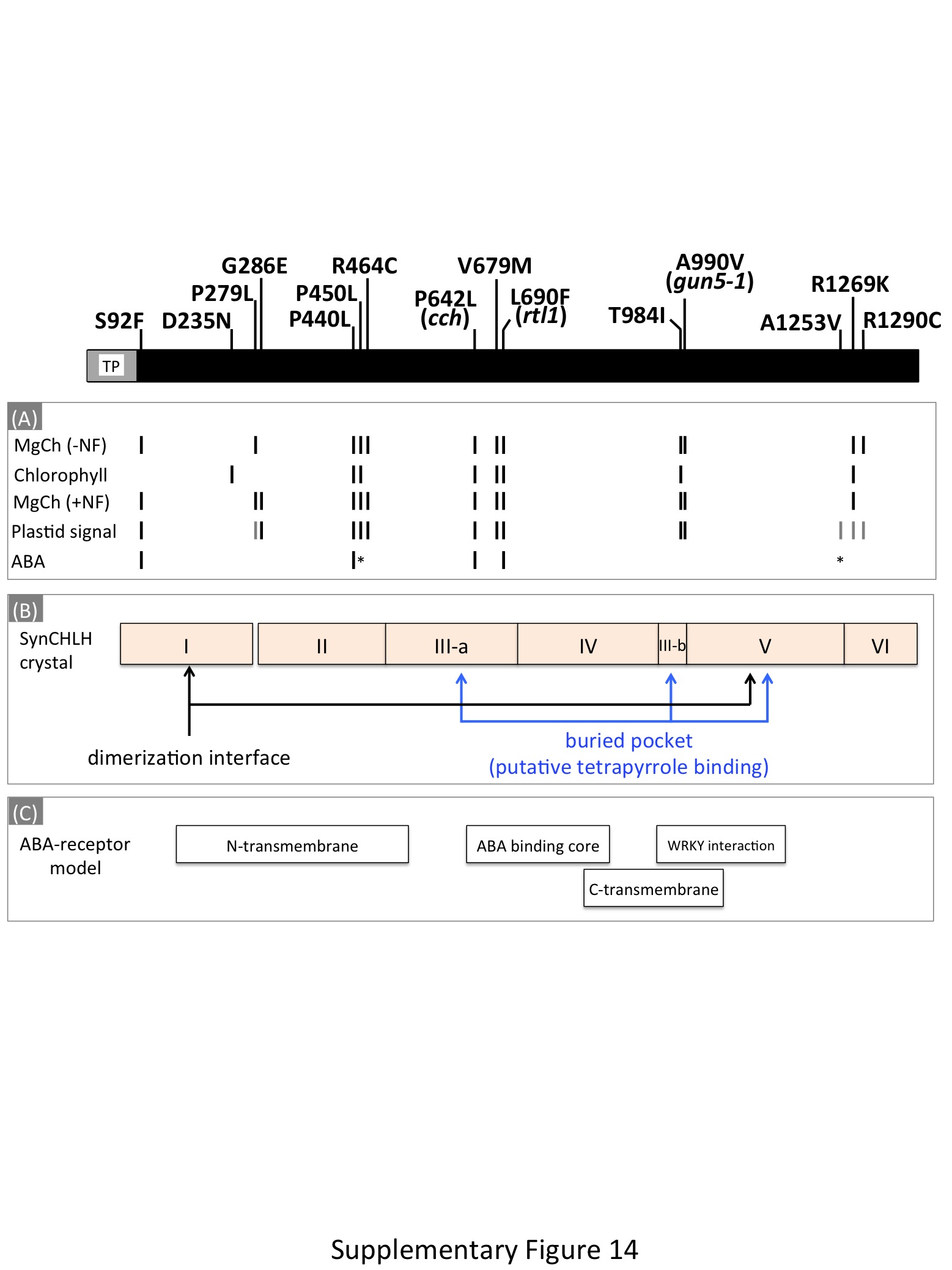

Supplement: Supplementary Figure 14 — Schematic diagrams of the currently proposed functional and structural domain architecture of the CHLH protein, and the positions of the amino acid substitutions and phenotypes in the gun5 mutants used in this study. (Top) Black and gray boxes represent the CHLH protein and the transit peptide, respectively. Wild type amino acids and positions and mutant amino acids are indicated. (A) Vertical black and gray lines represent mutants showing strong or moderate phenotypes in the assays indicated in the left, respectively. Asterisks indicate the mutants exhibiting constitutively closed stomatal phenotypes. (B) Green boxes represent the proposed domain structures based on crystallography analysis of SynCHLH (Chen et al., 2015). (C) White boxes represent the proposed regions responsible for ABA binding, the transmembrane domain and the WRKY interaction domain (Wu et al., 2009; Shang et al., 2010). [file Image14.JPEG]
