# Supplementary material for: CHLH/GUN5 Function in Tetrapyrrole Metabolism Is Correlated with Plastid Signaling but not ABA Responses in Guard Cells
Source: Front Plant Sci. 2016 Nov 7;7:1650. doi: 10.3389/fpls.2016.01650 (PMC5098175; doi:10.3389/fpls.2016.01650)
Supplement: Supplementary Table 1 — Primers used in this study. [file Table1.pdf]

Supplementary Table 1 Primers used in this study.

Name	sequence (5' to 3')	Purpose
CAB3pro-Fw0	GCTAGCGCCTGTGTCATTGGTGTGTGG	Lhcb1*2 promoter cloning
CAB3pro-Rv0'	TTGTGGAGGCGGCCATGGAAACTTTTTGTG	
CHLHpro-SL-1	TGCCTGCAGGTCCCCTTCTGCAGTAGGCGTTAATC	CHLH promoter cloning
CHLHp3R	ACAAGAAGGTACCATTTTGCGGCTGCTGGATTCTCC	
pGWB514-SL-11	GGGGACCTGCAGGCATGCAAGCTTGGCACT	
GW500spacerF	ATGGTACCTTCTTGTACAAAGTGGTT	
Forward primers for tGUN5s		
CHLH-SL-met1	AGCAGCCGCAAAATGGCTTCGCTTGTGTATTCTCCATTC	Fw primer for full, A, B, C, D, E, F, G
CHLH-SL-121F	AGCAGCCGCAAAATGGACAAGAACACTTACAACAACTTCTGCGAA	Fw primer for H
CHLH-SL-311F	AGCAGCCGCAAAATGGACACCAATGACTCACTCAAGAGG	Fw primer for I
CHLH-SL-631F-2	AGCAGCCGCAAAATGCCCATGAGGCTGCTTTTCTCC	Fw primer for J
Reverse primers for tGUN5s		
CHLH-SL-1381R	ACTTTGTACAAGAAGCGATCGATCCCTTCGATCTTGTC	Rv primer for full, E, H, I, J
CHLH-SL-1303R	ACTTTGTACAAGAAGCTCCATCCCACAGTGTTGGACAGTCTC	Rv primer for full, E, H, I, JA, F
CHLH-SL-999-R	ACTTTGTACAAGAAGGTTGTGGGAATAGCCTGAGGA	Rv primer for full, E, H, I, JB, G
CHLH-SL-772-R2	ACTTTGTACAAGAAGTCAAGATTACATTGCTTAGCTGTGCTGATG	Rv primer for full, E, H, I, JC
CHLH-SL-630-R	ACTTTGTACAAGAAGTCACCTTCATACCCAAATGTTGGTTG	Rv primer for full, E, H, I, JD
CHLH-GW-Met	CACCATGGCTTCGCTTGTGTATTCTCCATTCACTC	E fragment production by overlapping PCR
CHLH-120_631−Rv	GCAGCCTCATTCTAAGCTCCTCGACCAAGTA	
CHLH-120_631-Fw	GGAGCTTAGAATGAGGCTGCTTTTCTCCAAG	
CHLH-1381-Rv	TCGATCGATCCCTTCGATCTTGTC	
CHLH F51	ACATACTGAGGGAAGCTCGCAG	Amplification of GUN5 genomic region
CHLH Rv5	ATCATCCAAAGAACCTGCCC	
CHLH Fw5	CGATGGAGCCAATCGTTTTC	
CHLH R30	GCACTTCAGCTTCAGAAAGCGG	
CHLH Fw1	ATGGCTTCGCTTGTGTATTCTCC	DNA sequencing of GUN5 genomic region
CHLH Fw3	GTTAAGGATGCGGTGGAGAA	
CHLH Fw4	GGTATGACACTAGAAGGGAC	
CHLH Fw5	CGATGGAGCCAATCGTTTTC	
CHLH Fw6	GAGAGAACCTTCTTGTCTATGG	
CHLH Fw7	GTCCACAGATCGTCAGTTCC	
CHLH Fw8	CTTACCAACCGGAAAAACATCC	
CHLH Fw9	GGCATTGATATTAGAGAGGCAG	
CHLH Fw10	CAACACCAAGTTCTACAGGG	
CHLH Fw11	CGAAGGAATGATGTCAAGTGG	
CHLH Fw12	CTACCAAACGACATCATCTCTC	
CHLH Fw13	CCGCAAATACGATCTTCAAGAGAGCAAC	
CHLH Rv1	CTCTTGTGGAGAGCATGTGACT	
CHLH Rv4	GGGAATGTTCCCGATAAGACTG	
CHLH Rv5	ATCATCCAAAGAACCTGCCC	
CHLH R56	GGATGTATATCCGCTTGGGACAATGAG	
CHLH-Z1-F	GGAATTCTATGGCTTCGCTTGTGTATTCTC	CHLH antibody epitope production
CHLH-Z1-R	ACGCGTCGACTTATCGATCGATCCCTTCGATCTTG	
TUB 2/3 Fw1	CCAGCTTTGGTGATTTGAAC	qPCR primers
TUB 2/3 Rv1	CAAGCTTTCGGAGGTCAGAG	
CAB3 RT FW	GTGTGACAATGAGGAAGACTGTTGCC	
CAB3 RT RV	AAATGCTCTGAGCGTGGACCAAGCTA	
CHLH Fw9	GGCATTGATATTAGAGAGGCAG	
CHLH Rv6	GACGAGTTTTCAACAGCAAGAC	
